# Comparison of outpatient attendance, cardiovascular risk management and cardiovascular health across preCOVID-19, during and postCOVID-19 periods: a prospective cohort study

**DOI:** 10.1136/bmjopen-2024-092374

**Published:** 2025-07-16

**Authors:** Anna G M Zondag, Saskia Haitjema, Mark C H de Groot, Annemarijn R de Boer, Wouter W van Solinge, Michiel L Bots, Robin W M Vernooij

**Affiliations:** 1 Central Diagnostic Laboratory, Utrecht University, University Medical Centre Utrecht, Utrecht, The Netherlands; 2 Julius Center for Health Sciences and Primary Care, Utrecht University, University Medical Centre Utrecht, Utrecht, The Netherlands; 3 Department of Nephrology and Hypertension, University Medical Centre Utrecht, Utrecht, The Netherlands

**Keywords:** COVID-19, Cardiovascular Disease, Health Services Accessibility, EPIDEMIOLOGY

## Abstract

**Abstract:**

**Objective:**

During the COVID-19 pandemic, a substantial decrease was observed in hospital admissions and in-hospital procedures for patients with acute cardiovascular diseases (CVDs). The extent to which measures to prevent COVID-19 transmission, for example, lockdowns, affected the outpatient care of patients at higher cardiovascular risk remains unclear. We aimed to compare outpatient department (OPD) attendance, cardiovascular risk management (CVRM) and cardiovascular health (CVH) of patients at higher cardiovascular risk referred to an OPD of a tertiary care centre between preCOVID-19, during and postCOVID-19 periods.

**Design, setting and participants:**

We included all adult patients at higher cardiovascular risk referred to the cardiology, vascular medicine, diabetology, geriatrics, nephrology or multidisciplinary vascular surgery OPDs of the University Medical Centre Utrecht, the Netherlands, between March 2019 and December 2022, in a prospective cohort study.

**Main outcome measures:**

We assessed trends in the number of first and follow-up appointments and in the completeness of extractable CVRM indicators from the electronic health record (EHR) as a proxy for CVRM guideline adherence. CVH was determined using the Life’s Essential 8 metric (score 0–100, the higher score, the better). We investigated whether CVH differed between COVID-19 periods compared with the reference period (ie, 2019) and stratified by OPDs, using multivariable linear regression, adjusted for age, gender, CVD history and whether the patient had a previous appointment before the reference period.

**Results:**

Among 15 143 patients, we observed a 33% reduction in the weekly number of first appointments during the COVID-19 pandemic, with the largest reductions in the cardiology and nephrology OPDs, with no differences between women and men. Follow-up appointments conducted remotely, compared with before the COVID-19 pandemic, increased significantly for all OPDs. CVRM indicators were up to 11% less extractable during the first lockdown yet returned to prepandemic levels directly after the first lockdown period. The CVH score of patients visiting the nephrology, vascular medicine and geriatrics OPDs during the first lockdown was 11.23 (95% CI 2.74 to 19.72), 5.68 (95% CI 0.82 to 10.54) and 5.66 (95% CI 0.01 to 11.31) points higher, respectively, compared with the prepandemic period. In between the second and third lockdowns, the CVH score was comparable to the preCOVID reference period, yet for the cardiology OPD it was significantly higher (5.54, 95% CI 2.04 to 9.05).

**Conclusions:**

During the COVID-19 pandemic, weekly numbers of first appointments to OPDs decreased, and a population with a higher CVH score (ie, better CVH) visited certain OPDs, especially during the first lockdown period. These suggest that patients with poorer CVH more often avoided or were unable to visit OPDs, which might have resulted in missed opportunities to control cardiovascular risk factors and potentially may have led to preventable disease outcomes. For future epidemics and pandemics, it seems vital to develop a strategy that includes an emphasis on seeking healthcare when needed, with specific attention to patients at higher CVD risk.

**STRENGTHS AND LIMITATIONS OF THIS STUDY**

- This study is among the first to assess trends in outpatient department visits, cardiovascular risk management factor registration and cardiovascular health (CVH) during the COVID-19 pandemic compared with a reference period in 2019.
- The use of routine care data in this study allowed us to get insight into real-world practices during the COVID-19 pandemic.
- Although CVH estimates for a population based on the Life’s Essential 8 scoring metric had to be adjusted slightly due to unavailable information on passive smoking, diet, physical activity and sleep in the electronic health records, it clearly demonstrated its usability in providing an impression of the health of the population.

## Introduction

 The COVID-19 pandemic had a direct impact on morbidity and mortality worldwide, as well as an indirect impact on patients with or at risk of cardiovascular diseases (CVDs). To reduce the risk of transmission, governmental policies, such as social distancing measures, home quarantines and mobility restrictions (ie, lockdowns), were adopted.^1 2^ In the Netherlands, between March and June 2020, the first lockdown was implemented, and several others followed.^3^ In healthcare, routine diagnostic appointments related to diseases other than COVID-19 were cancelled, postponed or transformed into digital consultations to limit the risk of COVID-19 transmission and to prioritise the care of patients with COVID-19.^4 5^

Previous studies have found a substantial reduction in the number of hospital admissions and in-hospital procedures for patients with acute CVD when comparing the numbers during the first COVID-19 wave to a comparable period in 2019.^6^,^9^ Similar trends were observed in the Netherlands, with an 8% reduction in the total number of hospital admissions for CVD when comparing 2020 to 2019, potentially due to fear of acquiring a COVID-19 infection in hospitals.^10^ Patients who experienced postponed care were more likely to have poorer (self-reported) health and chronic diseases.^11^ Whether this resulted in a different patient population using healthcare services during COVID-19 lockdowns, in terms of clinical characteristics, remains to be investigated, as no comparison was made to a prepandemic group.

To date, less research has focused on the extent to which the periods of lockdown and their associated measures to prevent transmission affected patients at higher cardiovascular risk (ie, with a CVD or a risk factor for CVD) attending outpatient departments (OPDs) in terms of OPD attendance, cardiovascular risk management (CVRM) and cardiovascular health (CVH). Postponing care, either by choice or by the healthcare provider, may have increased the risk of CVD (progression), specifically for this patient population who benefits from CVRM. Therefore, we aimed to investigate trends in OPD attendance, CVRM and CVH in patients at higher cardiovascular risk who were referred to an OPD of a tertiary care centre in the Netherlands by comparing preCOVID-19, during and postCOVID-19 pandemic periods.

## Methods

We used Strengthening the Reporting of Observational Studies in Epidemiology as the reporting guideline for this observational study.^12^

### Study design and population

We designed a prospective cohort study in which all adult patients (aged ≥18 years) who visited an OPD of the University Medical Centre (UMC) Utrecht for evaluation of a CVD or a CVD risk factor were eligible to participate. Patients attending the following OPDs were eligible: cardiology, vascular medicine, nephrology, diabetology, geriatrics and multidisciplinary vascular surgery. Of all patients visiting the geriatric OPD, only the patients visiting the general geriatric OPD or the OPD specific for falling or memory problems were eligible. We excluded patients from the nephrology OPD who had a kidney transplantation within 365 days before or after their first appointment, as these patients are not part of the population under nephrology care eligible for CVRM.

### Data collection and variables

We collected data from all eligible patients who visited the UMC Utrecht between March 2019 and December 2022 to cover periods before, during and after the COVID-19 pandemic. We used the Utrecht Patient Oriented Database (UPOD) to collect data from the electronic health records (EHRs).^13^ In short, the UPOD database comprises, among other things, demographic data, laboratory values, medical procedures and prescribed medication data of all patients who have visited the UMC Utrecht since 2004.^13^ We extracted data at baseline (ie, at the time of the patient’s first appointment) on age, biological gender, visiting OPD, CVD and CV risk factor history (eg, hypertension and diabetes), prescribed medication (Anatomical Therapeutic Chemical (ATC) coded), smoking status, Body Mass Index (BMI, kg/m^2^), systolic and diastolic blood pressure (mm Hg), heart rate (HR, bpm), total cholesterol (mmol/L), high-density lipoprotein cholesterol (HDL-c, mmol/L), low-density lipoprotein cholesterol (LDL-c, mmol/L), non-HDL-c (mmol/L), triglycerides (mmol/L), glycated haemoglobin (HbA1c, mmol/mol), haemoglobin (Hb, g/L) and creatinine (µmol/L). We estimated the glomerular filtration rate (eg, eGFR, ml/min/1.73 m^2^) using the new equation based on age, biological gender and serum creatinine without ethnicity, as this information is not available in the EHR.^14^ In addition, we collected the date, time and location of all follow-up appointments after the first appointment up until December 2022. Online supplemental 1, table A provides the source and definition of each variable used in this study. Apart from the disease-specific OPD, the reason for referral was not available.

For further insights into the severity of the COVID-19 situation during the COVID-19 pandemic, we used data on the SARS-CoV-2 virus load in Dutch sewage water, which was collected by the Dutch National Institute for Public Health and the Environment.^15^

### Determinant

**Figure 1:**
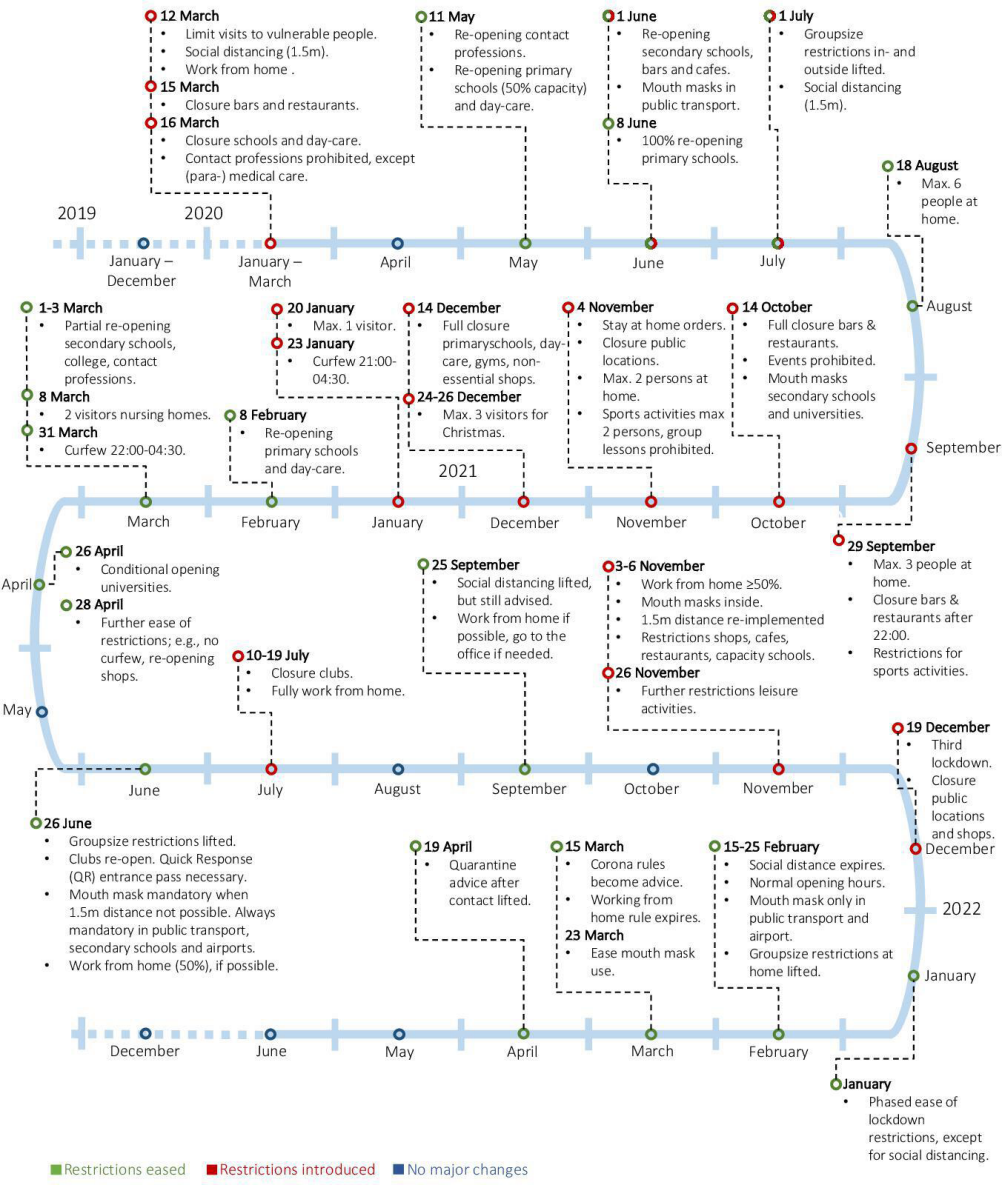
Summary of the coronavirus timeline and its restrictions, based on information from the Dutch National Institute for Public Health and the Environment.^3^

The periods between 2019 and 2022 during which several lockdowns were imposed were the main determinant in this study. We defined the periods based on dates on which restrictions and lockdowns were imposed in the Netherlands (table 1) using the ‘timeline of corona measures’, developed and updated by the Dutch National Institute for Public Health and the Environment throughout the COVID-19 pandemic.^3^ The timeline shows, in detail, the COVID-19 measures and restrictions imposed on specific dates between 2020 and 2023. Figure 1 illustrates the restrictions imposed in the Netherlands between 2020 and 2022, including the specific dates. During the COVID-19 pandemic, the role of the Dutch National Institute for Public Health and the Environment was, among other things, to monitor and report on the spread of the disease, to provide scientific advice to the government on possible measures to reduce transmission and to issue public health guidelines.^16^

**Table 1:** Period definition, corresponding dates and the length of each period in weeks

Period	Date range	Length (in weeks)
Reference (before COVID-19 pandemic)	11 March 2019–7 July 2019	17
Period before first lockdown	8 July 2019–8 March 2020	35
First lockdown	9 March 2020–5 July 2020	17
Postfirst lockdown	6 July 2020–11October 2020	14
Second lockdown	12 October 2020–2 May 2021	29
Postsecond lockdown	3 May 2021–19 December 2021	33
Third lockdown	20 December 2021–30 January 2022	6
PostCOVID-19 pandemic	31 January ’22–31 December ‘22	48

### Statistical analysis

#### Baseline characteristics

Baseline characteristics at the time of the first appointment in the hospital were summarised and presented descriptively as means and SD, medians and IQRs or numbers and percentages, as appropriate, and presented by period, overall and stratified by OPD.

#### OPD attendance

To analyse trends in OPD attendance, we categorised all appointments as a first or follow-up appointment, using the first appointment as the baseline. Subsequent appointments, regardless of the OPD but restricted to the cardiology, vascular medicine, diabetology, geriatrics, multidisciplinary vascular surgery and nephrology OPDs, were considered follow-up appointments and categorised as either in person or remote (ie, by telephone or video), as indicated in the description of the appointment code. We visualised the total number of first appointments per week over time and calculated the average SARS-CoV-2 virus load per week in Dutch sewage water to provide more information about the prevalence of the disease over time. Then, we calculated the mean number of first appointments per week per period by dividing the total number of first appointments per period by the length of the period (in weeks) and visually assessed whether the trend differed between genders. We additionally presented these trends stratified by OPD, as we assumed that the effect of the COVID-19 pandemic on the number of first-time appointments differed between OPD. Finally, we calculated and visualised the mean number of follow-up appointments per 100 patients per period and by OPD to assess differences over time.

#### CVRM guideline adherence

We determined the extractability from structured fields of the EHR at baseline for CVRM indicators that should be assessed regularly in all patients at higher cardiovascular risk: BMI, smoking status, blood pressure, HR, serum lipids, HbA1c, Hb and kidney function (ie, eGFR).^17^ Except for smoking, we considered an indicator to be extractable if registered within ±21 days from the date of the first appointment in a structured designated field of the EHR. Smoking status was extractable if registered in the designated field of the EHR ±6 months from the date of the first appointment. We presented the extractability of CVRM indicators in percentages by period. Using multivariable logistic regression, we tested for effect modification by gender and OPD for all CVRM indicators to assess whether trends over time differed between gender and OPD. In both cases, we performed an analysis of deviance to determine the overall significance of the interaction term. We considered a two-sided α≤0.05 to be statistically significant.

#### CVH between periods

We assessed the patient’s CVH using the American Heart Association’s (AHA) Life’s Essential 8 (LE8), which scores eight metrics to determine CVH, namely, diet, nicotine exposure, sleep, blood lipids, blood glucose, physical activity, BMI and blood pressure.^18^ In routine care, data on sleep, diet and physical activity are commonly not collected in structured fields of the EHR. Therefore, we assessed CVH based on five metrics instead of eight. We calculated the total score by summing the available metrics scored and then dividing the sum by the number of available metrics per patient. The scoring process of the metrics used in our study can be found in online supplemental 2, table B. We performed multivariable linear regression analyses to assess whether the moment of the first visit (ie, during which specific period before, during or after the COVID-19 pandemic) was associated with CVH. We adjusted for biological gender, age and whether the patient had had a previous appointment (before the reference period in 2019). We further tested if gender or OPD modified this association. In case of effect modification, stratified results were presented.

As a sensitivity analysis, we used multiple imputation using the mice package in R with default settings (eg, five multiple imputations and five iterations) to impute the missing data stratified by OPD. After imputation, we repeated the multivariable linear regression analyses to compare the CVH score of patients visiting the OPDs during the COVID-19 pandemic with patients visiting the OPDs during the reference period in 2019, before the start of the COVID-19 pandemic. We compared the results with the results from the unimputed data.

All analyses were conducted using R Studio and R V.4.0.5.^19^

### Patient and public involvement

Patients and/or the public were not involved in the design, conduct, reporting or dissemination plans of this research.

## Results

### Baseline patient characteristics

**Figure 2:**
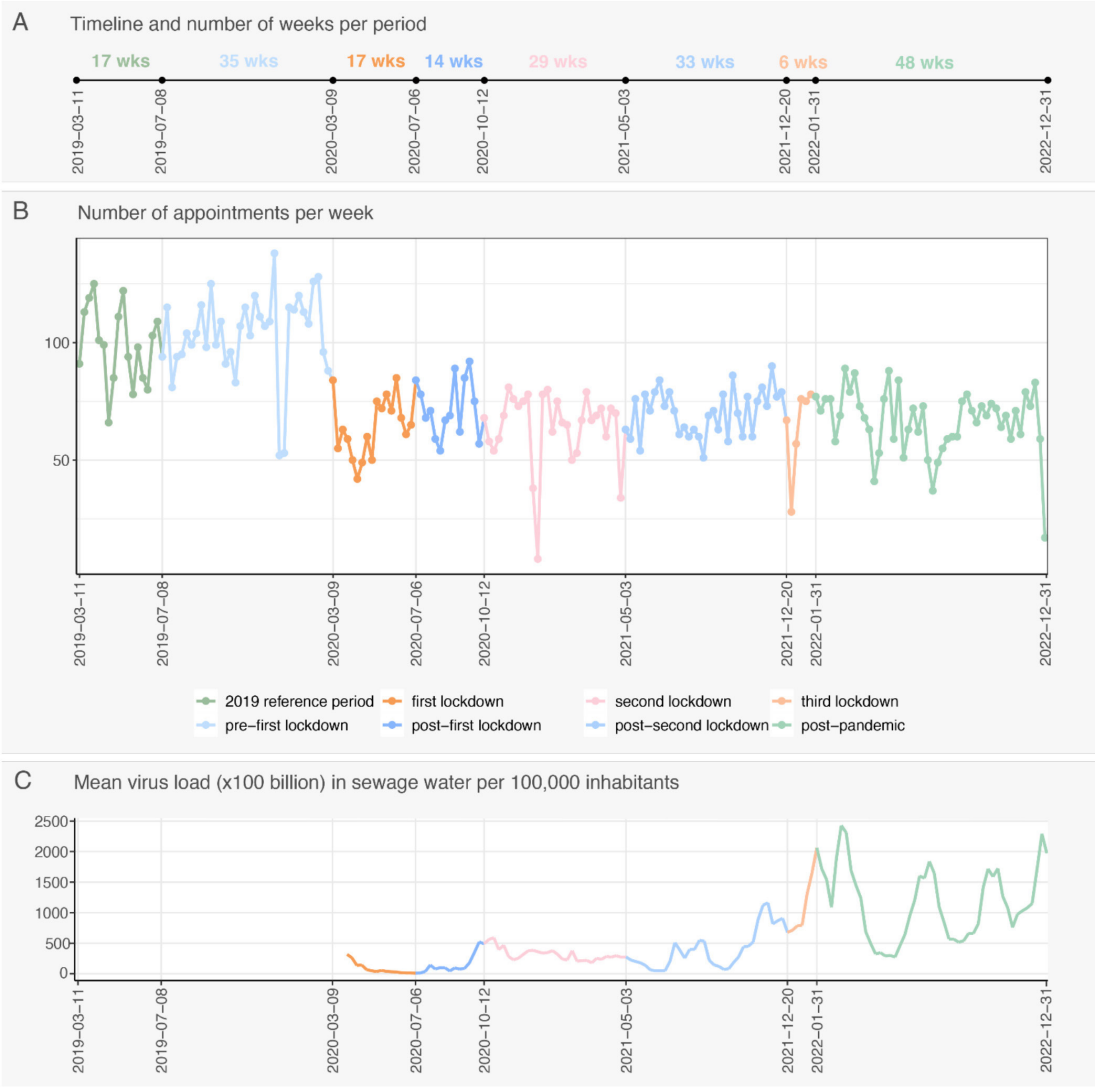
Total number of first appointments per week (B), including an illustration of the length of the periods (A) and the SARS-CoV-2 virus load over time (C). Data source for C: National Institute for Public Health and the Environment.^15^

In total, 15 143 patients were included in this study. The number of patients per period varied, partly because the periods were not of equal length (figure 2A). The median age was 59 years, and 50.5% were women (table 2). Most cardiovascular indicators had a similar baseline distribution across the COVID-19 periods. Patients who visited the hospital during the first lockdown and the period thereafter had a higher median eGFR (95.7 and 94.2 mL/min/1.73 m^2^, respectively) compared with patients seen during the reference period (89.5 mL/min/1.73 m^2^). Furthermore, patients visiting the OPDs during the second lockdown until the end of the COVID-19 pandemic more often had a history of hypertension, diabetes and CVD than patients visiting the OPDs in the reference period.

**Table 2:** Baseline patient characteristics, by period

	Reference period, n=1679	Period before first lockdown, n=3626	First lockdown, n=1087	Postfirst lockdown, n=1010	Second lockdown, n=1856	Postsecond lockdown, n=2313	Third lockdown, n=381	Postpandemic, n=3191
Gender (female), n (%)	824 (49.1)	1832 (50.5)	530 (48.8)	546 (54.1)	938 (50.5)	1170 (50.6)	196 (51.4)	1607 (50.4)
Age, median (IQR)	60.0 (43.0–71.0)	61.0 (46.0–71.0)	58.0 (40.0–70.0)	59.0 (40.0–71.0)	59.0 (44.0–71.0)	59.0 (41.0–71.0)	59.0 (39.0–72.0)	59.0 (40.0–71.0)
Smoker (yes), n (%)	77 (42.3)	154 (35.2)	46 (43.8)	40 (35.4)	84 (38.5)	101 (41.1)	21 (43.8)	127 (46.0)
BMI, median (IQR)	26.2 (23.4–29.4)	26.2 (23.4–29.6)	26.2 (23.2–29.9)	25.5 (23.0–29.3)	26.2 (23.3–29.5)	25.8 (22.9–29.4)	26.7 (23.4–30.8)	25.9 (23.2–29.2)
sysBP, mean (SD)	137.8 (25.0)	141.0 (25.8)	137.1 (25.3)	137.3 (24.5)	141.8 (26.4)	138.8 (25.6)	139.0 (24.1)	138.8 (26.2)
HR, mean (SD)	75.1 (15.0)	75.5 (16.4)	74.5 (14.7)	73.0 (15.3)	75.3 (14.9)	75.4 (16.0)	76.0 (13.2)	74.4 (14.8)
Hb, mean (SD)	136.5 (18.4)	137.3 (17.5)	138.8 (18.1)	138.2 (19.8)	140.5 (18.9)	139.5 (19.8)	136.0 (20.4)	135.4 (19.1)
Tot-c, median (IQR)	5.0 (4.2–6.0)	4.7 (4.0–5.7)	4.8 (4.0–5.6)	4.7 (3.8–5.5)	4.7 (4.0–5.7)	4.6 (3.9–5.5)	4.6 (3.9–5.6)	4.6 (3.8–5.5)
HDL-c, median (IQR)	1.3 (1.1–1.6)	1.3 (1.1–1.6)	1.3 (1.1–1.7)	1.3 (1.1–1.6)	1.3 (1.1–1.6)	1.2 (1.0–1.5)	1.2 (1.0–1.5)	1.3 (1.0–1.6)
LDL-c, median (IQR)	2.9 (2.1–3.7)	2.5 (1.9–3.3)	2.5 (1.9–3.2)	2.6 (1.8–3.2)	2.6 (1.9–3.3)	2.5 (1.9–3.2)	2.6 (1.9–3.3)	2.5 (1.9–3.3)
Trig, median (IQR)	1.5 (1.1–2.3)	1.6 (1.1–2.2)	1.5 (1.1–2.2)	1.4 (1.0–2.1)	1.5 (1.0–2.1)	1.6 (1.1–2.4)	1.6 (1.1–2.4)	1.4 (1.0–2.1)
HbA1c, median (IQR)	40.0 (36.0–49.0)	39.0 (36.0–46.0)	39.0 (35.0–44.8)	39.0 (35.0–45.0)	39.0 (35.0–48.0)	38.0 (35.0–45.0)	38.0 (34.0–47.5)	38.0 (34.0–44.0)
eGFR (CKD-EPI), median (IQR)	89.5 (66.8–104.7)	91.4 (71.1–104.1)	95.7 (82.3–108.9)	94.2 (74.5–110.8)	92.5 (70.7–107.4)	90.3 (68.5–107.0)	91.2 (71.1–107.5)	90.5 (69.2–106.6)
CVD history (yes), n (%)	231 (13.8)	478 (13.2)	148 (13.6)	144 (14.3)	325 (17.5)	370 (16.0)	60 (15.7)	523 (16.4)
Hyp history (yes), n (%)	43 (2.6)	108 (3.0)	33 (3.0)	32 (3.2)	73 (3.9)	80 (3.5)	19 (5.0)	123 (3.9)
Diab (yes), n (%)	150 (8.9)	296 (8.2)	87 (8.0)	78 (7.7)	174 (9.4)	235 (10.2)	37 (9.7)	301 (9.4)

BMI, body mass index; CVD, cardiovascular disease; Diab, diabetes; eGFR (CKD-EPI), estimated glomerular filtration rate calculated using the chronic kidney disease epidemiology collaboration equation; Hb, haemoglobin; HbA1c, glycated haemoglobin; HDL-c, high-density lipoprotein cholesterol; HR, heart rate; Hyp, hypertension; LDL-c, low-density lipoprotein cholesterol; n, number; sysBP, systolic blood pressure; Tot-c, total cholesterol; Trig, triglycerides.

Some differences are visible between OPDs (online supplemental 3, tables C–H). For example, patients visiting the diabetology, nephrology and vascular medicine OPDs during the first lockdown and the period after the first lockdown were younger and had a higher eGFR compared with patients in the prepandemic reference period. Lower systolic blood pressure (sysBP) was found in patients visiting the diabetes and vascular medicine OPDs.

### OPD attendance

#### First appointments over time, overall and by OPD

Overall, there was a decrease in the number of first appointments from the first lockdown onwards, which did not return to prepandemic levels (figure 2B). Prepandemic (ie, during the reference and the prefirst lockdown periods), the median number of first appointments per week was 99 (IQR 85–111) and 107 (IQR 95–115), respectively, while during the COVID-19 pandemic, the median number of first appointments ranged between 63 and 71 (online supplemental 4, table I).

**Figure 3:**
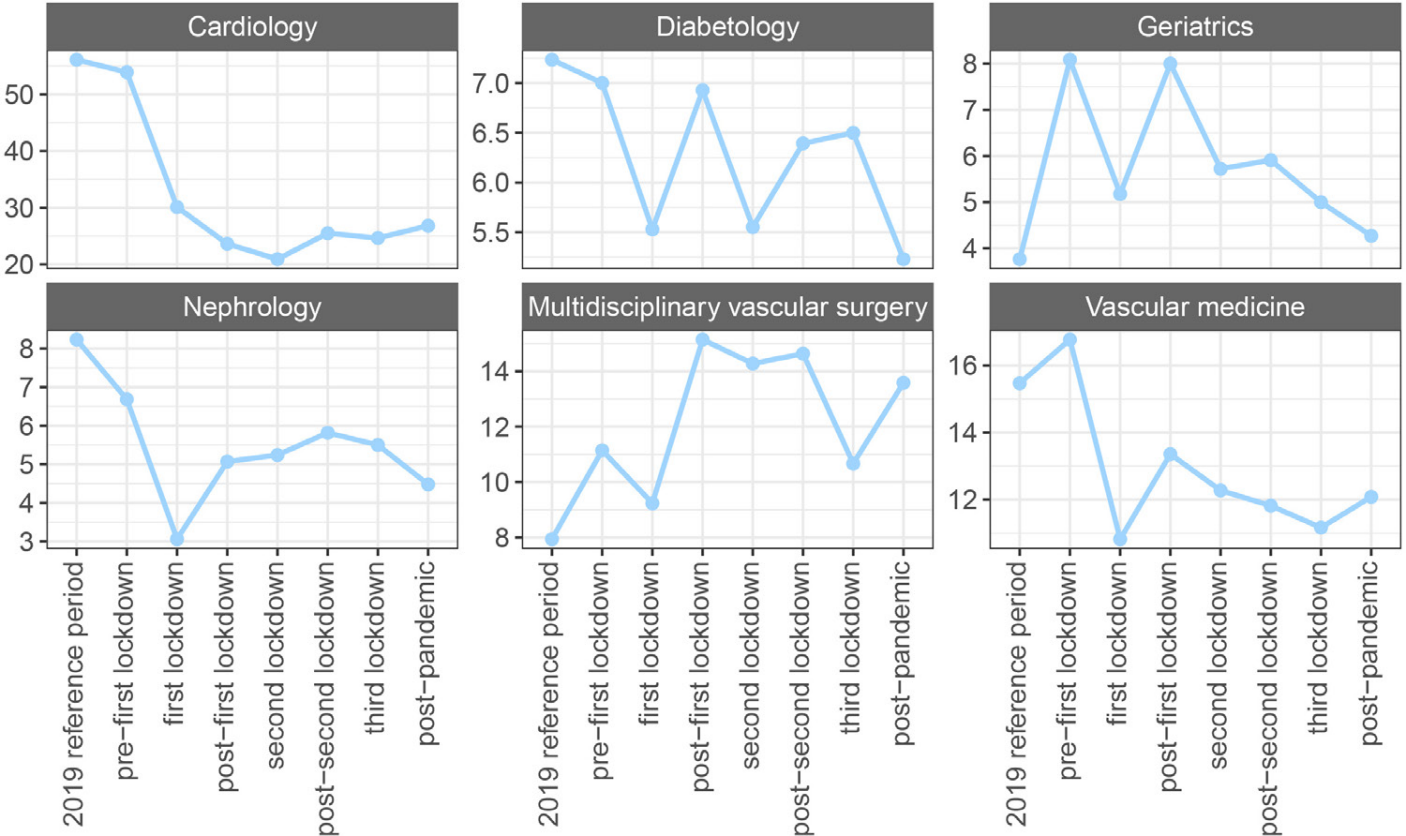
Total number of first appointments across periods, divided by the length of the COVID-19 period (in weeks), stratified by outpatient department (OPD).

The trend in the number of first appointments across periods did not differ between genders but did differ between OPDs (figure 3). Except for the multidisciplinary vascular surgery and geriatrics OPDs, a decline in the number of first appointments per period was visible from the moment of the first lockdown onwards. For the geriatrics and multidisciplinary vascular surgery OPDs, the number of first appointments during the COVID-19 pandemic remained higher than during the reference period.

#### Follow-up appointments over time, overall and by OPD

**Figure 4:**
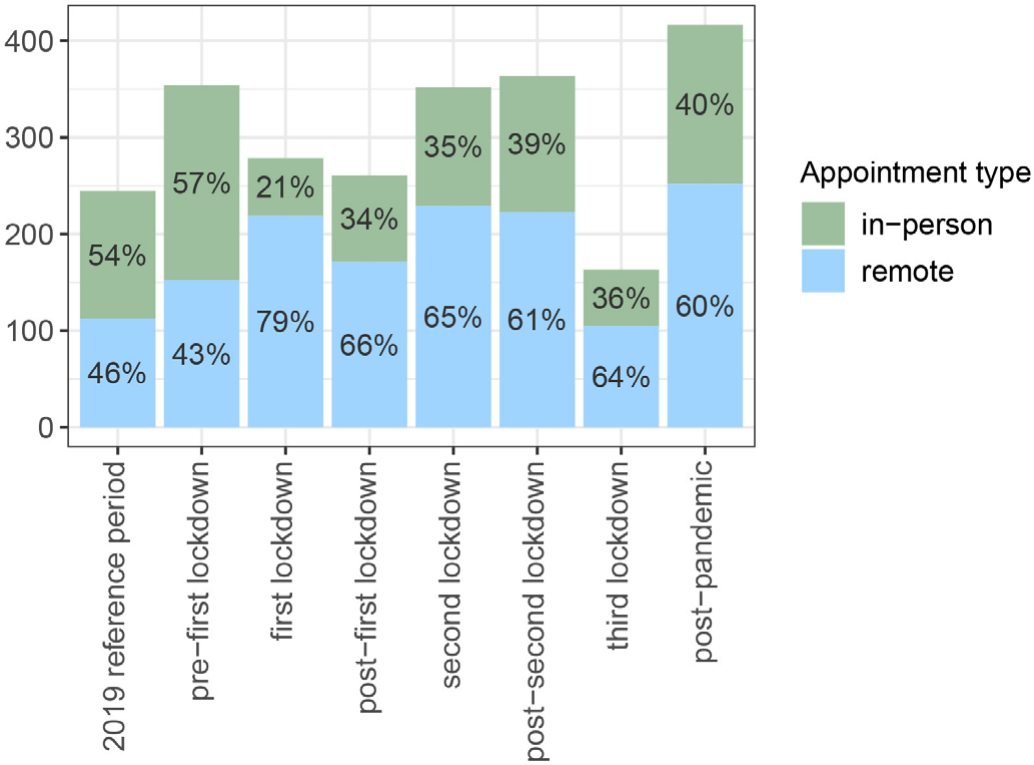
Mean number of follow-up appointments per 100 patients, by type of appointment.

While, in general, we observed a decrease in the number of first appointments, the mean number of follow-up appointments (either in person or remote) per 100 patients remained comparable across periods (ie, the reference and prefirst lockdown periods vs the periods during the COVID-19 pandemic), except during the third lockdown, when the number was lower than during the reference period (figure 4). Prepandemic (ie, during the reference period and the period before the lockdown), most follow-up appointments took place in person (54%–57%, respectively). When the COVID-19 pandemic started, remote formats (ie, by phone or video) were widely adopted, with up to 79% of all follow-up appointments in some periods. At the end of the COVID-19 pandemic (ie, postpandemic period), the percentage of remote follow-up appointments remained high (60% vs 46%–43%).

Trends were similar between OPDs; however, the uptake of remote follow-up appointments differed (online supplemental 5, figure A). For example, most follow-up appointments in the geriatric OPD took place in person. Nevertheless, when comparing the first lockdown to the reference period, the percentage of remote appointments doubled. In all other OPDs, most of the follow-up appointments took place remotely. Both the cardiology and vascular medicine OPDs were the only OPDs where >50% of the follow-up appointments were already conducted remotely before the COVID-19 pandemic.

#### CVRM guideline adherence

**Figure 5:**
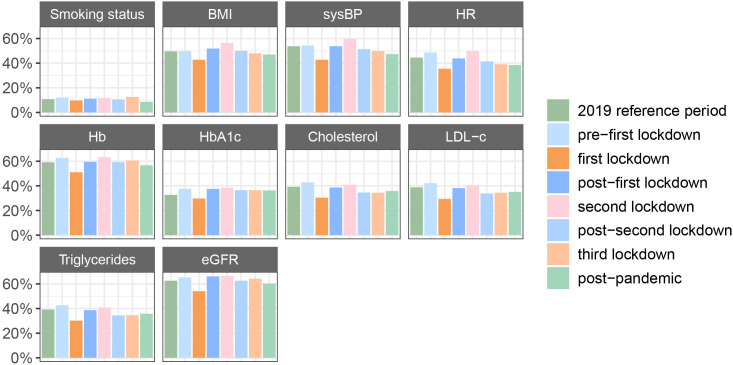
Extractability of CVRM indicators over time. BMI, body mass index; CVRM, cardiovascular risk management; eGFR, estimated glomerular filtration rate using the chronic kidney disease epidemiology collaboration equation; Hb, haemoglobin; HbA1c, glycated haemoglobin; HR, heart rate; LDL-c, low-density lipoprotein cholesterol and sysBP, systolic blood pressure.

Compared with the reference period, CVRM indicators were up to 11% less extractable during the first lockdown (figure 5, online supplemental 6, table J). The largest decrease between these periods was observed in the extractability of sysBP (−11%) and serum lipids (ie, cholesterol and triglycerides; −9%/−10%). Overall, the extractability of CVRM indicators, used as a proxy for guideline adherence, was low during all periods (ie, below ~60%) and not substantially different from the prepandemic situation.

Except for HDL-c, the association between the time period and the extractability of the CVRM indicators did not differ between genders (p≥0.05 for all interactions; online supplemental 6, table J). The association between the time period and the CVRM indicator extractability, however, significantly differed between OPDs (p<0.01 for all interactions; online supplemental 6, table J and figure B). In most OPDs, a drop in extractability is visible during the first lockdown. The geriatric OPD, however, remained relatively stable except after the second lockdown and during the third lockdown. During these periods, a substantial decrease was observable in the extractability of the laboratory measurements.

#### Association between the COVID-19 period and CVH of the patients

The results were stratified by OPD, as OPD modified the association between the period of the patient’s first appointment and CVH. Gender did not modify the relation. During the first lockdown, the CVH score of patients visiting the vascular medicine, nephrology and geriatrics OPDs was, respectively, on average 5.68 (95% CI 0.82 to 10.54), 11.23 (95% CI 2.74 to 19.72) and 5.66 (95% CI 0.01 to 11.31) points higher than that of patients who visited these OPDs during the reference period in 2019 (table 3). Between the second and third lockdowns, the CVH score was comparable to the preCOVID reference period, yet for the cardiology OPD, the CVH score significantly increased (5.54, 95% CI 2.04 to 9.05). The sensitivity analysis, in which we accounted for the missing data using multiple imputation, yielded similar results (online supplemental 7, table K).

**Table 3:** Association between period of the first appointment and CVH, adjusted for covariates,* stratified by OPD

	Cardiology	Vascular medicine	Nephrology	Geriatrics	Diabetology	Multidisciplinary vascular surgery
	Beta (95% CI)	Beta (95% CI)	Beta (95% CI)	Beta (95% CI)	Beta (95% CI)	Beta (95% CI)
Intercept	77.87 (73.85 to 81.91)	78.14 (73.68 to 82.60)	75.37 (68.80 to 81.94)	60.04 (50.68 to 69.40)	86.93 (81.89 to 91.97)	67.20 (60.33 to 74.08)
COVID-19 period						
Reference period	Ref.	Ref.	Ref.	Ref.	Ref.	Ref.
Prefirst lockdown	2.01 (−0.81 to 4.84)	4.91 (1.40 to 8.42)	−2.54 (−7.88 to 2.80)	−0.01 (−4.78 to 4.75)	3.13 (−1.44 to 7.70)	5.05 (−0.40 to 10.49)
First lockdown	−1.08 (−5.42 to 3.26)	5.68 (0.82 to 10.54)	11.23 (2.74 to 19.72)	5.66 (0.01 to 11.31)	4.77 (−0.98 to 10.52)	1.86 (−4.95 to 8.67)
Postfirst lockdown	3.30 (−1.48 to 8.09)	3.63 (−0.92 to 8.18)	6.39 (−1.17 to 13.96)	3.45 (−2.00 to 8.90)	4.14 (−1.43 to 9.71)	1.19 (−4.98 to 7.36)
Second lockdown	0.25 (−3.60 to 4.10)	0.95 (−2.88 to 4.79)	−2.40 (−8.27 to 3.46)	3.48 (−1.61 to 8.58)	−0.60 (−5.53 to 4.32)	−0.84 (−6.24 to 4.56)
Postsecond lockdown	5.54 (2.04 to 9.05)	−0.44 (−4.31 to 3.44)	1.61 (−3.90 to 7.13)	2.78 (−2.19 to 7.76)	2.82 (−1.87 to 7.50)	4.77 (−0.62 to 10.17)
Third lockdown	3.26 (−3.32 to 9.83)	1.74 (−4.92 to 8.40)	1.44 (−8.29 to 11.17)	0.19 (−7.40 to 7.77)	−5.23 (−12.81 to 2.35)	−4.27 (−13.14 to 4.61)
Postpandemic	3.16 (−0.01 to 6.33)	3.97 (0.42 to 7.52)	4.53 (−0.89 to 9.95)	6.50 (1.56 to 11.44)	5.41 (0.86 to 9.95)	4.89 (−0.43 to 10.20)

The green cells indicate a significant increase in CVH score (ie, better CVH).

* Adjusted for age, biological gender, CVD history and whether the patient had an appointment before the study period.

beta, beta coefficient indicating the difference in CVH score between the periods during the COVID-19 pandemic and the reference period; CVD, cardiovascular disease; CVH, cardiovascular health; OPD, outpatient department.

## Discussion

### Summary of the findings

In summary, patients with or at high risk of CVD were still seen at the OPDs during the COVID-19 pandemic, but to a lesser extent than in the prepandemic reference period, depending on the OPD. In all OPDs, we observed a substantial uptake of remote formats for follow-up appointments. Overall, the extractability of all CVRM indicators, used as a proxy for CVRM guideline adherence, decreased during the first lockdown yet rebounded afterwards. This differed slightly between OPDs. Patients who visited the vascular medicine, geriatric or nephrology OPDs during the first lockdown had a significantly higher CVH score, indicating better CVH, compared with patients in the reference period. Similarly, the patients visiting the cardiology OPD in the period right after the second lockdown had a higher CVH score.

### Explanation and comparison with existing literature

Studies with different study populations have examined the utilisation of outpatient care services during the COVID-19 pandemic. For instance, one study investigated the use of outpatient services without restriction on the type of care (eg, primary or specialty),^20^ or investigated the visits to any medical institution in an outpatient setting in Korea,^21^ while another study specifically examined visits to the cardiology outpatient clinic.^22^ Consistent with these studies, we found a substantial drop in the number of first-time visits to the OPD when the first lockdown was introduced.^20^,^22^ The explanation is potentially multifactorial. In the Netherlands, to make an appointment at an OPD of any hospital, one needs to be referred by a general practitioner (GP) or by secondary care. The number of referrals from GPs to hospitals decreased significantly (from 100 000 referrals the week before the first lockdown to, at worst, 26 000 per week during the first lockdown), especially the referrals for non-acute plannable care.^23^ However, after the first COVID-19 wave, this rebounded to prepandemic levels, a rebound that we did not observe in the actual number of first appointments to OPDs in our study. There may be several reasons for the sustained reduction in first appointments. It could be due to healthcare providers being requested to stay at home in case of any COVID-19 symptoms,^3^ which may have led to staff shortages as healthcare providers were at significantly greater risk of a COVID-19 infection.^24^ This is supported by other studies that found a significant increase in sick leave related to infectious diseases among healthcare workers during the pandemic.^25 26^ Additionally, patients were advised to stay at home as much as possible, especially the elderly and frail who were at higher risk of a worse COVID-19 prognosis. Hence, people may have avoided healthcare facilities, both GP offices and OPDs, altogether out of fear of infection.^4 27^ It is also likely that not all OPD appointments were urgent. Patients who tested positive for COVID-19 would, therefore, have been advised not to attend their appointment. Also, the patients eligible for our study, that is, patients with CVD or a CVD risk factor (eg, hypertension and diabetes), were at higher risk of a worse COVID-19 disease prognosis, which could be an additional reason for them not attending their OPD appointment.

Furthermore, measures adopted in hospitals may also have influenced the OPD capacity. Healthcare in the Netherlands was restructured around the 1.5-metre social distancing restriction. According to a report published in 2020 by, among others, the Dutch Society of Cardiology, the adaptations necessary to adhere to social distancing restrictions would lead to an estimated reduction in the regular capacity of cardiology OPDs of between 40% and 60%.^28^ This estimated reduction is broadly in line with our findings, with an overall decrease of ~30% in the median number of appointments from the first lockdown onwards, compared with corresponding periods in 2019. However, if we look at each OPD individually, the cardiology OPD experienced a ~58% decrease in the number of first appointments. A smaller decrease (ie, in the nephrology, diabetology and vascular medicine OPDs) or no decrease (ie, in the geriatrics and multidisciplinary vascular surgery OPDs) was observed in the other OPDs. This, together with the potential reasons mentioned earlier, could explain the number of first appointments remaining low during the rest of the time periods. We did, however, not find a decrease in follow-up appointments, potentially due to the quick uptake of telemedicine (ie, the remote follow-up appointments in our study conducted by phone or video). The rapid increase of telemedicine was also found in other studies.^29 30^ The less substantial increase in remote follow-up appointments in the geriatric OPD found in our study is not surprising and can be explained by the fact that patients receiving geriatric care are often older and frailer, potentially making telemedicine more challenging due to limited digital literacy.

Karalis *et al*^31^ compared adherence to the 2017 guideline for high blood pressure and the 2018 American College of Cardiology/AHA/Multisociety guideline in the management of cholesterol during the COVID-19 pandemic with a prepandemic period in patients with CVD visiting cardiology outpatient clinics for secondary prevention in the USA. In line with our results, they found a drop in guideline adherence during the first lockdown as compared with the reference period,^31^ which could be explained by the sudden disruption and changes in healthcare, during which COVID-19-related urgent healthcare interventions were prioritised over non-urgent care. It may also be that the uncertainties in the early days of the COVID-19 pandemic about the routes of transmission and how best to protect oneself from infection may have led both clinicians and patients to be more cautious and, therefore, to have close contact only when deemed necessary, resulting in social distancing in outpatient clinics. As a result, fewer measurements requiring physician interaction, such as laboratory measurements, may have been performed and registered in EHRs. The lack of these CVRM indicators might affect optimal CVRM in clinical practice, as less information on the patient’s CVD risk profile is available. For example, to predict a patient’s 10-year risk of CVD morbidity and mortality using cardiovascular risk algorithms, such as the SMART risk score, the patient’s lipid profile is needed.^32 33^ When the indicator is missing, the calculator will impute the missing indicator using the population mean, affecting the accuracy of the prediction.^32^

Furthermore, during specific periods of the COVID-19 pandemic, we observed that the patients attending the OPDs were generally healthier in terms of CVH than patients attending the OPDs during the reference period before the COVID-19 pandemic. This suggests that a selection of the regular patient population visited the hospital during these periods. Although the point difference in CVH compared with the reference group may seem small, it is worth noting that the risk of all-cause and CVD mortality is 14% and 19% lower, respectively, per 10-point increase in CVH score.^34^ Therefore, our findings may indicate that patients at higher risk of a (cardiovascular) disease event visited the outpatient clinics less often, which may have led to preventable disease outcomes.^11^

For future epidemics and pandemics, it seems vital to develop a strategy that includes an emphasis on seeking healthcare when needed, with specific attention to patients at higher risk. Furthermore, further study is warranted to evaluate how remote appointments may have influenced the patient’s prognosis. Equally important would be to study whether patients with worse CVH, who avoided or were unable to attend OPDs, indeed more often suffered from events that could have been prevented.

### Limitations

Limitations of our study must be considered when interpreting our results. First, the data source of all variables in this study is routine care data collected from structured fields of our EHR. In routine care, oftentimes, physicians only perform measurements or order laboratory tests that contribute to decision-making. Thus, the absence of an indicator could be meaningful and may not be completely random.^35^ This did not affect our findings in terms of hospital appointments and extractability but may have affected our findings regarding the comparison of the patient populations visiting the OPDs across the different periods. Second, we adjusted the scoring metric of the LE8, as not all information was available from the EHR (ie, information on passive smoking, sleep, physical activity and diet). However, this unlikely influenced our results since Howard *et al*^36^ showed that the CVH scores calculated using the less complex Life’s Simple 7, which does not include passive smoking and sleep, were highly correlated with the CVH scores calculated using LE8. Third, although COVID-19 was a global pandemic, our study focused exclusively on its impact within a tertiary care centre in the Netherlands. The extrapolation of our results to other countries may be challenging, given the variability of the COVID-19 pandemic situation and the restrictive measures imposed across countries and between healthcare settings. Finally, it is debatable whether the periods that we have defined based on the coronavirus timeline developed by the Dutch National Institute for Public Health and the Environment are comparable, as, for example, seasonality plays a role in the number of appointments (eg, less appointments during national holiday periods such as Christmas). We have tried to mitigate these potential influences on our results by at least having a reference period in 2019 that is the same period of the year as the period of the first lockdown in 2020, to ensure that these periods were truly comparable.

### Conclusions

During the COVID-19 pandemic, the weekly number of first appointments to OPDs decreased, and most follow-up appointments were conducted remotely. Based on CVRM extractable information, guideline adherence remained largely unaffected over time. A population with a higher CVH score visited certain OPDs during the COVID-19 pandemic, specifically in the first lockdown period, suggesting that patients with poorer CVH more often avoided or were unable to visit OPDs. This might have resulted in missed opportunities to control cardiovascular risk factors, which may potentially have led to preventable disease outcomes.

## Supplementary material

10.1136/bmjopen-2024-092374 online supplemental file 1

10.1136/bmjopen-2024-092374 online supplemental file 2

10.1136/bmjopen-2024-092374 online supplemental file 3

10.1136/bmjopen-2024-092374 online supplemental file 4

10.1136/bmjopen-2024-092374 online supplemental file 5

10.1136/bmjopen-2024-092374 online supplemental file 6

10.1136/bmjopen-2024-092374 online supplemental file 7

## Data Availability

Data are available upon reasonable request.

## References

[1] Ebrahim SH, Ahmed QA, Gozzer E (2020). Covid-19 and community mitigation strategies in a pandemic. BMJ.

[2] Meyerowitz-Katz G, Bhatt S, Ratmann O (2021). Is the cure really worse than the disease? The health impacts of lockdowns during COVID-19. BMJ Glob Health.

[3] RIVM Timeline of coronavirus measures (in Dutch only).

[4] Kiss P, Carcel C, Hockham C (2021). The impact of the COVID-19 pandemic on the care and management of patients with acute cardiovascular disease: a systematic review. Eur Heart J Qual Care Clin Outcomes.

[5] Rosell Ortiz F, Fernández Del Valle P, Knox EC (2020). Influence of the Covid-19 pandemic on out-of-hospital cardiac arrest. A Spanish nationwide prospective cohort study. Resuscitation.

[6] Rinkel LA, Prick JCM, Slot RER (2021). Impact of the COVID-19 outbreak on acute stroke care. J Neurol.

[7] Neves Briard J, Ducroux C, Jacquin G (2021). Early Impact of the COVID-19 Pandemic on Acute Stroke Treatment Delays. Can J Neurol Sci.

[8] Tam C-CF, Cheung K-S, Lam S (2020). Impact of Coronavirus Disease 2019 (COVID-19) Outbreak on ST-Segment-Elevation Myocardial Infarction Care in Hong Kong, China. Circ Cardiovasc Qual Outcomes.

[9] Mafham MM, Spata E, Goldacre R (2020). COVID-19 pandemic and admission rates for and management of acute coronary syndromes in England. Lancet.

[10] Koop Y, Wimmers RH, Vaartjes I (2021). Cardiovascular diseases in the Netherlands, 2021 (in Dutch Only).

[11] Visscher K, Kouwenberg LHJA, Oosterhoff M (2023). Postponed healthcare in The Netherlands during the COVID-19 pandemic and its impact on self-reported health. *Front Health Serv*.

[12] von Elm E, Altman DG, Egger M (2008). The Strengthening the Reporting of Observational Studies in Epidemiology (STROBE) statement: guidelines for reporting observational studies. J Clin Epidemiol.

[13] ten Berg MJ, Huisman A, van den Bemt PMLA (2007). Linking laboratory and medication data: new opportunities for pharmacoepidemiological research. Clin Chem Lab Med.

[14] Inker LA, Eneanya ND, Coresh J (2021). New Creatinine- and Cystatin C-Based Equations to Estimate GFR without Race. N Engl J Med.

[15] RIVM (2024). Weekly coronavirus SARS-CoV-2 figures. Rijksinitituut voor Volksgezondheid en Milieu. https://www.rivm.nl/en/coronavirus-covid-19/current/weekly-update.

[16] Infectious Disease Control National Institute for Public Health and the Environment.

[17] Visseren FLJ, Mach F, Smulders YM (2021). 2021 ESC Guidelines on cardiovascular disease prevention in clinical practice. Eur Heart J.

[18] Lloyd-Jones DM, Allen NB, Anderson CAM (2022). Life’s Essential 8: Updating and Enhancing the American Heart Association’s Construct of Cardiovascular Health: A Presidential Advisory From the American Heart Association. *Circulation*.

[19] R Core Team (2021). R: A Language and Environment for Statistical Computing. R Foundation for Statistical Computing.

[20] Dupraz J, Le Pogam M-A, Peytremann-Bridevaux I (2022). Early impact of the COVID-19 pandemic on in-person outpatient care utilisation: a rapid review. BMJ Open.

[21] Sim B, Nam EW (2022). The Impact of COVID-19 Pandemic on Outpatient Visits for All-Cause and Chronic Diseases in Korea: A Nationwide Population-Based Study. Int J Environ Res Public Health.

[22] Wosik J, Clowse MEB, Overton R (2021). Impact of the COVID-19 pandemic on patterns of outpatient cardiovascular care. Am Heart J.

[23] Giessen A, Wit A, Brink C (2020). Impact of the first COVID-19 wave on regular healthcare and care (summary in English only).

[24] Nguyen LH, Drew DA, Graham MS (2020). Risk of COVID-19 among front-line health-care workers and the general community: a prospective cohort study. Lancet Public Health.

[25] Reme B-A, Grøsland M, Gjefsen H (2023). Impact of the COVID-19 pandemic on sick leave among healthcare workers: a register-based observational study. Occup Environ Med.

[26] Calvo-Bonacho E, Catalina-Romero C, Fernández-Labandera C (2020). COVID-19 and Sick Leave: An Analysis of the Ibermutua Cohort of Over 1,651,305 Spanish Workers in the First Trimester of 2020. Front Public Health.

[27] Splinter MJ, Velek P, Ikram MK (2021). Prevalence and determinants of healthcare avoidance during the COVID-19 pandemic: A population-based cross-sectional study. PLoS Med.

[28] Gupta Strategists, NVVC, Medtronic (2020). The new normal for regular care in a corona era (in Dutch only).

[29] Patel SY, Mehrotra A, Huskamp HA (2021). Trends in Outpatient Care Delivery and Telemedicine During the COVID-19 Pandemic in the US. JAMA Intern Med.

[30] Uscher-Pines L, Sousa J, Jones M (2021). Telehealth Use Among Safety-Net Organizations in California During the COVID-19 Pandemic. JAMA.

[31] Karalis DG, Moeller P, Crawford A (2023). Impact of the COVID-19 pandemic on the management of risk factors in patients with stable atherosclerotic vascular disease. *Am J Prev Cardiol*.

[32] Dorresteijn JAN, Visseren F (2021). The SMART Risk Score. Europ Soc Cardiol.

[33] U-Prevent (2024). U-Prevent: You are in control.

[34] Yi J, Wang L, Guo X (2023). Association of Life’s Essential 8 with all-cause and cardiovascular mortality among US adults: A prospective cohort study from the NHANES 2005-2014. Nutr Metab Cardiovasc Dis.

[35] Overmars LM, Niemantsverdriet MSA, Groenhof TKJ (2022). A Wolf in Sheep’s Clothing: Reuse of Routinely Obtained Laboratory Data in Research. J Med Internet Res.

[36] Howard G, Cushman M, Blair J (2024). Comparative Discrimination of Life’s Simple 7 and Life’s Essential 8 to Stratify Cardiovascular Risk: Is the Added Complexity Worth It?. Circulation.

