# Supplementary material for: Comparison of outpatient attendance, cardiovascular risk management and cardiovascular health across preCOVID-19, during and postCOVID-19 periods: a prospective cohort study
Source: BMJ Open. 2025 Jul 16;15(7):e092374. doi: 10.1136/bmjopen-2024-092374 (PMC12273069; doi:10.1136/bmjopen-2024-092374)
Supplement: online supplemental file 1 [file bmjopen-15-7-s001.pdf]

Supplement 1

Table A. Data source and definition per variable.

Variable	Source and definition
Demographic data (i.e., age and sex)	Based on data from structured fields of the EHR.
Hospital appointments (i.e., date, time, type of appointments, OPD)	All hospital appointments made after the first appointment, at specific OPDs: cardiology, geriatrics, diabetology, nephrology, vascular medicine and at the multidisciplinary vascular surgery.
Smoking status	Based on a structured questionnaire available for completion in the EHR for all patients within 6 months of the first appointment.
CVD history*	Cardiovascular event history, which included stroke, peripheral artery disease, coronary heart disease, and abdominal aortic aneurysm. Determined based on hospital procedures, billing codes, medical diagnosis registered in the EHR before the date of the first appointment.
Risk factors (e.g., hypertension, diabetes mellitus)	Based on registered medical diagnosis in the EHR before the date of the first appointment and prescribed medication.
Measurements<br>- Physical measurements: heart rate, BMI, systolic and diastolic blood pressure.<br>- Laboratory measurements: lipids, glycated haemoglobin, haemoglobin, creatinine	Extracted from structured fields in the EHR.<br>Within 21 days before or after the appointment dates.
Medication	All prescribed cardiovascular (risk factor) medication* in use at the time of the appointment, based on structured fields in the EHR.<br><br>Medication start date should be the same as the appointment date, or, if medication start date is before date of the appointment, then medication stop date should be after date of the appointment.<br><br>*ATC codes: A10, B01, B02, C01 until C10.

Notes: *Determined using an in-hospital developed and validated algorithm. EHR = electronic health record; CVD = cardiovascular disease; BMI = body mass index; ATC = anatomical therapeutic chemical classification.
