# Supplementary material for: Comparison of outpatient attendance, cardiovascular risk management and cardiovascular health across preCOVID-19, during and postCOVID-19 periods: a prospective cohort study
Source: BMJ Open. 2025 Jul 16;15(7):e092374. doi: 10.1136/bmjopen-2024-092374 (PMC12273069; doi:10.1136/bmjopen-2024-092374)
Supplement: online supplemental file 2 [file bmjopen-15-7-s002.pdf]

Supplement 2

Table B. Cardiovascular health scoring metric, adapted from AHA's LE8.[18]

Metric	Method of measurement	Quantification	
Nicotine exposure	Routine care data	Points	Status
		100	Never smoker
		75	Former smoker, quit ≥5 year
		50	Former smoker, quit 1–<5 year
		25	Former smoker, quit <1 year
		0	Current smoker
BMI	Routine care measurement	Points	Level
		100	<25
		70	25.0–29.9
		30	30.0–34.9
		15	35.0–39.9
		0	≥40.0
Blood lipids (non-HDL cholesterol, mg/dL)	Routine care measurement	Points	Level
		100	<130
		60	130–159
		40	160–189
		20	190–219
		0	≥220
		20 points were subtracted if the patient used or was prescribed lipid lowering medication (ATC code C10).	
Blood glucose (HbA1c, %)	Routine care measurement	Points	Level
		100	HbA1c <5.7
		60	HbA1c 5.7–6.4
		40	HbA1c 6.5–6.9
		30	HbA1c 7.0–7.9
		20	HbA1c 8.0–8.9
		10	HbA1c 9.0–9.9
		0	HbA1c ≥10.0
Blood pressure (mmHG)	Routine care measurement	Points	Level
		100	<120 systolic and <80 diastolic
		75	120–129 systolic and <80 diastolic
		50	130–139 systolic or 80–89 diastolic
		25	140–159 systolic or 90–99 diastolic
		0	≥160 systolic or ≥100 diastolic
		20 points were subtracted if the patient used any kind of blood pressure lowering medication (ATC codes C02, C03, C07, C08, C09).	

Notes: BMI = body mass index; non-HDL = total cholesterol minus high-density lipoprotein cholesterol.
