# Supplementary material for: Comparison of outpatient attendance, cardiovascular risk management and cardiovascular health across preCOVID-19, during and postCOVID-19 periods: a prospective cohort study
Source: BMJ Open. 2025 Jul 16;15(7):e092374. doi: 10.1136/bmjopen-2024-092374 (PMC12273069; doi:10.1136/bmjopen-2024-092374)
Supplement: online supplemental file 3 [file bmjopen-15-7-s003.pdf]

Supplement 3

Table C. Baseline patient characteristics of patients who visited the cardiology OPD, by COVID-19 period.

	**Reference period, n=954**	**period before 1st lockdown, n=1887**	**1st lockdown, n=512**	**Post-1st lockdown, n=331**	**2nd lockdown, n=607**	**Post-2nd lockdown, n=842**	**3rd lockdown, n=148**	**Post pandemic, n=1288**
Sex (female), n(%)	460 (48.2)	913 (48.4)	236 (46.1)	176 (53.2)	295 (48.6)	402 (47.7)	64 (43.2)	597 (46.4)
Age, median(IQR)	60.0 (45.0-71.0)	60.0 (45.0-70.0)	58.0 (38.0-67.2)	56.0 (36.0-68.0)	58.0 (40.0-70.0)	58.0 (37.0-69.0)	61.0 (41.0-70.0)	58.0 (38.0-71.0)
Smoker (yes), n(%)	37 (43.5)	79 (42.9)	23 (59.0)	9 (36.0)	22 (41.5)	26 (44.8)	7 (58.3)	49 (51.6)
BMI, median(IQR)	26.0 (23.5-28.7)	26.0 (23.1-29.2)	26.2 (23.2-30.0)	25.0 (22.4-28.1)	24.9 (22.4-28.7)	24.8 (22.5-28.7)	27.1 (23.1-32.3)	25.8 (23.3-29.0)
sysBP, mean(SD)	135.0 (22.4)	135.9 (22.1)	131.4 (22.9)	134.6 (24.6)	134.9 (24.4)	129.4 (21.3)	132.9 (17.2)	130.7 (21.3)
HR, mean(SD)	75.7 (16.5)	78.2 (19.5)	77.8 (17.8)	76.5 (15.5)	79.9 (18.7)	79.5 (20.6)	78.6 (14.1)	77.4 (17.2)
Hb, mean(SD)	137.2 (18.3)	137.8 (18.0)	136.9 (19.8)	139.3 (21.1)	139.2 (20.4)	139.4 (21.6)	136.0 (24.6)	137.1 (20.1)
Total cholesterol, median(IQR)	5.1 (4.2-6.0)	4.7 (3.9-5.6)	4.9 (4.3-5.7)	4.6 (3.7-5.4)	4.7 (3.8-5.5)	4.6 (3.9-5.3)	4.2 (3.7-5.0)	4.5 (3.7-5.5)
HDL-c, median(IQR)	1.4 (1.1-1.6)	1.3 (1.1-1.6)	1.3 (1.1-1.7)	1.3 (1.1-1.6)	1.3 (1.1-1.6)	1.2 (1.0-1.5)	1.1 (0.9-1.5)	1.2 (1.0-1.4)
LDL-c, median(IQR)	3.0 (2.2-3.8)	2.5 (1.9-3.3)	2.6 (2.1-3.4)	2.5 (1.8-3.1)	2.6 (1.9-3.3)	2.3 (1.9-3.1)	2.4 (1.8-2.9)	2.5 (1.9-3.4)
Triglycerides, median(IQR)	1.5 (1.0-2.1)	1.5 (1.0-2.1)	1.5 (1.1-2.1)	1.6 (1.0-2.3)	1.4 (1.0-2.0)	1.7 (1.1-2.4)	1.2 (1.0-1.8)	1.6 (1.1-2.4)
HbA1c, median(IQR)	39.0 (36.0-47.0)	38.0 (36.0-43.0)	38.0 (36.0-42.8)	39.0 (36.0-45.0)	39.0 (35.0-46.8)	39.0 (36.0-45.0)	36.0 (35.2-37.0)	38.0 (35.0-45.0)
eGFR (CKD-EPI), median(IQR)	89.9 (70.7-101.3)	93.0 (76.2-103.7)	97.1 (84.4-108.1)	95.9 (84.5-108.7)	92.5 (70.4-105.2)	90.7 (71.3-104.5)	89.6 (70.3-99.7)	91.2 (69.6-105.1)
CVD history (yes), n(%)	102 (10.7)	168 (8.9)	34 (6.7)	21 (6.3)	49 (8.1)	62 (7.4)	11 (7.4)	100 (7.8)
Hypertension history (yes), n(%)	4 (0.4)	9 (0.5)	4 (0.8)	1 (0.3)	3 (0.5)	5 (0.6)	0 (0.0)	3 (0.2)
Diabetes (yes), n(%)	38 (4.0)	81 (4.3)	19 (3.7)	15 (4.5)	26 (4.3)	53 (6.3)	8 (5.4)	110 (8.5)

Notes: n = number; % = percentage; IQR = interquartile range; BMI = body mass index; sysBP = systolic blood pressure; HR = heart rate; SD = standard deviation; Hb = haemoglobin; HDL-c = high-density lipoprotein cholesterol; LDL-c = low-density lipoprotein cholesterol; HbA1c = glycated haemoglobin; eGFR CKD-EPI= estimated glomerular filtration rate using the chronic kidney disease epidemiology collaboration equation; CVD = cardiovascular disease.

Table D. Baseline patient characteristics of patients who visited the diabetology OPD, by COVID-19 period.

	**Reference period, n=123**	**period before 1st lockdown, n=245**	**1st lockdown, n=94**	**Post-1st lockdown, n=97**	**2nd lockdown, n=161**	**Post-2nd lockdown, n=211**	**3rd lockdown, n=39**	**Post pandemic, n=251**
Sex (female), n(%)	84 (68.3)	173 (70.6)	71 (75.5)	76 (78.4)	112 (69.6)	160 (75.8)	34 (87.2)	199 (79.3)
Age, median(IQR)	39.0 (32.0-56.5)	40.0 (31.0-61.0)	34.0 (29.0-49.0)	34.0 (30.0-39.0)	36.0 (30.0-50.0)	36.0 (31.5-49.5)	35.0 (29.0-42.5)	35.0 (30.0-51.0)
Smoker (yes), n(%)	1 (25.0)	10 (76.9)	2 (33.3)	4 (80.0)	9 (81.8)	10 (62.5)	1 (50.0)	4 (57.1)
BMI, median(IQR)	26.9 (24.4-30.2)	26.4 (23.4-30.8)	29.1 (23.5-33.4)	26.4 (22.7-30.9)	26.6 (24.5-31.8)	27.9 (22.9-31.6)	26.4 (24.6-32.5)	26.7 (23.5-30.8)
sysBP, mean(SD)	124.0 (18.6)	124.7 (21.3)	119.6 (15.3)	115.9 (13.5)	123.8 (18.9)	121.6 (18.3)	123.1 (16.7)	120.9 (16.6)
HR, mean(SD)	78.0 (11.5)	79.6 (15.5)	80.5 (10.0)	84.6 (10.9)	81.4 (14.9)	82.4 (13.3)	82.3 (8.7)	81.0 (13.4)
Hb, mean(SD)	132.8 (19.5)	132.0 (16.4)	131.4 (16.0)	130.8 (17.7)	134.0 (19.1)	131.1 (19.5)	127.2 (19.7)	126.9 (16.6)
Total cholesterol, median(IQR)	4.7 (4.0-5.4)	4.5 (3.9-5.4)	4.1 (3.3-4.9)	4.2 (3.7-5.1)	4.7 (4.1-5.1)	4.4 (3.9-5.1)	5.1 (4.3-5.5)	4.4 (3.7-5.4)
HDL-c, median(IQR)	1.2 (1.0-1.5)	1.3 (1.0-1.6)	1.3 (1.0-1.6)	1.2 (1.1-1.6)	1.2 (1.0-1.6)	1.3 (1.0-1.6)	1.4 (1.1-1.7)	1.2 (0.9-1.6)
LDL-c, median(IQR)	2.6 (1.9-3.2)	2.3 (1.6-3.0)	1.8 (1.6-2.4)	2.3 (1.7-3.2)	2.5 (1.9-3.0)	2.3 (1.8-2.7)	2.6 (2.3-3.1)	2.1 (1.7-2.8)
Triglycerides, median(IQR)	1.7 (1.1-2.4)	1.7 (1.3-2.4)	1.8 (1.1-2.6)	1.3 (0.8-2.2)	1.4 (1.0-2.3)	1.5 (1.0-2.4)	2.0 (1.2-2.7)	1.4 (1.0-2.5)
HbA1c, median(IQR)	48.0 (37.0-64.0)	45.5 (36.0-65.0)	39.0 (35.0-51.0)	38.0 (34.0-47.0)	45.0 (35.8-63.0)	40.0 (35.0-61.0)	39.0 (32.8-58.2)	38.0 (34.0-58.0)
eGFR (CKD-EPI), median(IQR)	117.4 (99.7-126.1)	114.2 (91.8-125.9)	125.3 (102.1-130.8)	126.8 (117.4-131.7)	122.9 (100.0-129.7)	121.2 (101.5-128.6)	119.5 (108.8-126.9)	121.8 (102.2-129.2)
CVD history (yes), n(%)	5 (4.1)	8 (3.3)	1 (1.1)	0 (0.0)	5 (3.1)	7 (3.3)	1 (2.6)	4 (1.6)
Hypertension history (yes), n(%)	0 (0.0)	1 (0.4)	0 (0.0)	0 (0.0)	2 (1.2)	1 (0.5)	0 (0.0)	1 (0.4)
Diabetes (yes), n(%)	59 (48.0)	103 (42.2)	34 (36.2)	31 (32.0)	83 (51.6)	88 (41.7)	12 (30.8)	103 (41.0)

Notes: n = number; % = percentage; IQR = interquartile range; BMI = body mass index; sysBP = systolic blood pressure; HR = heart rate; SD = standard deviation; Hb = haemoglobin; HDL-c = high-density lipoprotein cholesterol; LDL-c = low-density lipoprotein cholesterol; HbA1c = glycated haemoglobin; eGFR CKD-EPI = estimated glomerular filtration rate using the chronic kidney disease epidemiology collaboration equation; CVD = cardiovascular disease.

Table E. Baseline patient characteristics of patients who visited the geriatrics OPD, by COVID-19 period.

	**Reference period, n=64**	**period before 1st lockdown, n=283**	**1st lockdown, n=88**	**Post-1st lockdown, n=112**	**2nd lockdown, n=166**	**Post-2nd lockdown, n=195**	**3rd lockdown, n=30**	**Post pandemic, n=205**
Sex (female), n(%)	34 (53.1)	150 (53.0)	48 (54.5)	53 (47.3)	92 (55.4)	106 (54.4)	17 (56.7)	107 (52.2)
Age, median(IQR)	80.0 (73.0-82.0)	79.0 (72.0-83.0)	77.5 (71.0-83.2)	78.5 (72.0-84.0)	77.0 (71.0-82.0)	76.0 (71.5-82.0)	76.5 (73.2-80.8)	76.0 (70.0-82.0)
Smoker (yes), n(%)	6 (46.2)	13 (20.3)	2 (18.2)	6 (28.6)	1 (5.9)	13 (31.7)	5 (45.5)	8 (44.4)
BMI, median(IQR)	26.2 (22.5-30.5)	26.6 (23.8-29.7)	25.5 (22.9-29.7)	25.8 (23.6-29.4)	26.0 (23.2-28.2)	25.2 (22.6-28.4)	27.2 (23.2-29.5)	24.9 (23.0-28.1)
sysBP, mean(SD)	144.0 (24.5)	159.6 (23.2)	153.5 (25.1)	151.6 (20.2)	154.8 (25.1)	149.5 (22.2)	145.5 (18.2)	149.8 (24.7)
HR, mean(SD)	69.2 (9.2)	71.0 (12.8)	70.4 (12.0)	66.0 (15.6)	69.5 (11.6)	70.2 (13.1)	69.8 (12.6)	69.4 (13.0)
Hb, mean(SD)	134.2 (15.7)	137.4 (14.7)	139.1 (14.1)	137.9 (17.3)	140.4 (15.1)	139.9 (17.6)	134.6 (22.6)	134.5 (14.7)
Total cholesterol, median(IQR)	4.6 (4.0-5.5)	4.5 (3.9-5.5)	4.8 (3.9-5.5)	4.5 (3.6-5.1)	4.8 (4.1-5.6)	4.4 (3.8-5.1)	4.5 (3.9-5.2)	4.5 (3.9-5.4)
HDL-c, median(IQR)	1.3 (1.1-1.7)	1.3 (1.1-1.6)	1.5 (1.1-1.8)	1.3 (1.1-1.6)	1.4 (1.2-1.7)	1.3 (1.1-1.6)	1.2 (1.0-1.4)	1.4 (1.0-1.7)
LDL-c, median(IQR)	2.5 (2.0-3.2)	2.3 (1.7-3.0)	2.3 (1.9-2.8)	2.4 (1.8-2.9)	2.6 (2.0-3.2)	2.3 (1.8-3.1)	2.3 (1.9-3.1)	2.4 (1.8-3.2)
Triglycerides, median(IQR)	1.5 (1.2-2.3)	1.6 (1.2-2.3)	1.5 (1.0-2.0)	1.4 (1.1-1.9)	1.4 (0.9-1.8)	1.4 (1.0-1.8)	1.6 (1.5-2.2)	1.4 (1.0-1.9)
HbA1c, median(IQR)	42.0 (38.0-53.0)	40.0 (37.0-47.0)	40.0 (38.0-43.5)	41.0 (37.0-48.0)	40.0 (37.0-44.0)	39.0 (36.2-44.8)	40.0 (37.0-51.0)	39.0 (37.0-44.0)
eGFR (CKD-EPI), median(IQR)	73.6 (57.4-87.7)	79.7 (65.8-89.9)	85.0 (68.5-92.8)	81.4 (68.7-92.3)	85.3 (71.4-94.1)	82.6 (66.4-92.2)	77.6 (60.4-89.1)	82.4 (64.5-91.8)
CVD history (yes), n(%)	8 (12.5)	28 (9.9)	8 (9.1)	9 (8.0)	8 (4.8)	16 (8.2)	4 (13.3)	16 (7.8)
Hypertension history (yes), n(%)	0 (0.0)	3 (1.1)	1 (1.1)	1 (0.9)	1 (0.6)	2 (1.0)	0 (0.0)	1 (0.5)
Diabetes (yes), n(%)	10 (15.6)	29 (10.2)	10 (11.4)	9 (8.0)	9 (5.4)	19 (9.7)	3 (10.0)	18 (8.8)

Notes: n = number; % = percentage; IQR = interquartile range; BMI = body mass index; sysBP = systolic blood pressure; HR = heart rate; SD = standard deviation; Hb = haemoglobin; HDL-c = high-density lipoprotein cholesterol; LDL-c = low-density lipoprotein cholesterol; HbA1c = glycated haemoglobin; eGFR CKD-EPI = estimated glomerular filtration rate using the chronic kidney disease epidemiology collaboration equation; CVD = cardiovascular disease.

Table F. Baseline patient characteristics of patients who visited the nephrology OPD, by COVID-19 period.

	**Reference period, n=140**	**period before 1st lockdown, n=234**	**1st lockdown, n=52**	**Post-1st lockdown, n=71**	**2nd lockdown, n=152**	**Post-2nd lockdown, n=192**	**3rd lockdown, n=33**	**Post pandemic, n=215**
Sex (female), n(%)	57 (40.7)	113 (48.3)	23 (44.2)	34 (47.9)	67 (44.1)	90 (46.9)	18 (54.5)	95 (44.2)
Age, median(IQR)	63.0 (42.0-71.2)	61.0 (42.0-70.0)	59.0 (41.0-66.0)	60.0 (37.0-71.0)	55.0 (36.0-69.2)	60.5 (41.8-71.2)	50.0 (31.0-66.0)	59.0 (42.5-71.0)
Smoker (yes), n(%)	9 (36.0)	8 (42.1)	0 (0.0)	2 (33.3)	8 (53.3)	9 (52.9)	1 (100.0)	3 (42.9)
BMI, median(IQR)	26.3 (23.2-29.4)	27.0 (24.1-31.5)	27.0 (23.0-30.5)	24.0 (22.2-26.0)	27.0 (23.8-29.1)	26.4 (23.9-29.4)	26.8 (21.7-31.0)	25.9 (23.2-30.2)
sysBP, mean(SD)	141.0 (28.9)	142.1 (24.3)	137.4 (28.1)	137.1 (24.5)	141.9 (25.4)	138.8 (22.8)	132.0 (18.8)	140.7 (24.0)
HR, mean(SD)	75.7 (14.4)	76.6 (15.3)	73.4 (11.6)	73.8 (15.7)	76.1 (14.6)	75.9 (13.8)	75.5 (10.6)	73.9 (13.1)
Hb, mean(SD)	130.8 (19.7)	132.0 (19.1)	138.6 (17.9)	134.9 (19.6)	141.3 (18.8)	138.2 (19.0)	137.7 (11.0)	132.2 (18.9)
Total cholesterol, median(IQR)	5.2 (4.3-6.4)	5.0 (4.1-5.8)	4.8 (4.3-5.2)	4.8 (4.5-5.4)	5.1 (4.2-5.8)	4.9 (4.2-5.6)	5.3 (4.1-7.1)	4.8 (4.0-5.3)
HDL-c, median(IQR)	1.3 (1.0-1.6)	1.3 (1.1-1.6)	1.3 (1.0-1.4)	1.2 (1.0-1.5)	1.2 (1.1-1.6)	1.2 (1.0-1.5)	1.0 (1.0-1.4)	1.2 (1.0-1.6)
LDL-c, median(IQR)	3.0 (2.2-3.8)	2.8 (2.1-3.3)	2.7 (2.1-2.9)	2.8 (1.9-3.1)	2.7 (2.1-3.4)	2.6 (1.9-3.2)	3.1 (2.0-4.0)	2.4 (2.0-3.1)
Triglycerides, median(IQR)	1.9 (1.2-3.0)	1.8 (1.3-2.6)	1.9 (1.2-2.4)	1.6 (1.4-2.6)	1.8 (1.1-2.9)	1.9 (1.4-2.7)	2.0 (1.1-3.7)	1.6 (1.2-2.3)
HbA1c, median(IQR)	40.5 (35.2-46.8)	40.0 (35.0-49.0)	37.0 (34.0-40.2)	38.0 (34.0-41.0)	37.0 (35.0-40.0)	38.0 (35.0-43.0)	36.0 (33.0-40.0)	36.0 (34.0-41.0)
eGFR (CKD-EPI), median(IQR)	56.5 (37.0-90.4)	62.6 (44.2-93.7)	74.7 (56.1-99.9)	75.7 (47.9-107.1)	63.1 (41.9-93.2)	56.5 (38.1-82.7)	71.1 (48.0-111.7)	58.6 (40.0-89.2)
CVD history (yes), n(%)	14 (10.0)	18 (7.7)	4 (7.7)	7 (9.9)	12 (7.9)	14 (7.3)	3 (9.1)	12 (5.6)
Hypertension history (yes), n(%)	4 (2.9)	2 (0.9)	3 (5.8)	5 (7.0)	3 (2.0)	0 (0.0)	0 (0.0)	4 (1.9)
Diabetes (yes), n(%)	13 (9.3)	30 (12.8)	4 (7.7)	7 (9.9)	14 (9.2)	23 (12.0)	2 (6.1)	14 (6.5)

Notes: n = number; % = percentage; IQR = interquartile range; BMI = body mass index; sysBP = systolic blood pressure; HR = heart rate; SD = standard deviation; Hb = haemoglobin; HDL-c = high-density lipoprotein cholesterol; LDL-c = low-density lipoprotein cholesterol; HbA1c = glycated haemoglobin; eGFR CKD-EPI = estimated glomerular filtration rate using the chronic kidney disease epidemiology collaboration equation; CVD = cardiovascular disease.

Table G. Baseline patient characteristics of patients who visited the multidisciplinary vascular surgery OPD, by COVID-19 period.

	**Reference period, n=135**	**period before 1st lockdown, n=390**	**1st lockdown, n=157**	**Post-1st lockdown, n=212**	**2nd lockdown, n=414**	**Post-2nd lockdown, n=483**	**3rd lockdown, n=64**	**Post pandemic, n=652**
Sex (female), n(%)	42 (31.1)	157 (40.3)	62 (39.5)	90 (42.5)	170 (41.1)	208 (43.1)	28 (43.8)	295 (45.2)
Age, median(IQR)	65.0 (50.5-73.0)	67.0 (55.0-74.0)	65.0 (56.0-74.0)	64.0 (53.8-73.0)	65.0 (54.0-73.8)	65.0 (52.0-74.0)	66.0 (55.0-75.2)	65.0 (52.8-74.0)
Smoker (yes), n(%)	13 (56.5)	32 (47.8)	13 (52.0)	14 (42.4)	31 (45.6)	25 (43.9)	4 (33.3)	38 (52.8)
BMI, median(IQR)	25.9 (23.3-28.5)	25.9 (23.1-28.4)	25.4 (22.9-28.3)	25.6 (23.0-29.0)	26.1 (23.4-29.1)	25.0 (22.5-28.9)	25.8 (24.1-28.5)	25.1 (22.7-28.1)
sysBP, mean(SD)	139.9 (24.3)	139.9 (22.5)	139.0 (23.9)	139.7 (21.4)	141.8 (23.2)	139.2 (23.2)	152.9 (27.9)	142.6 (24.1)
HR, mean(SD)	75.0 (13.9)	73.8 (15.2)	74.8 (15.3)	71.8 (15.1)	75.0 (13.4)	73.6 (14.4)	75.7 (12.7)	73.5 (13.3)
Hb, mean(SD)	134.6 (19.1)	136.6 (18.8)	140.0 (15.6)	137.9 (21.0)	138.7 (19.7)	139.5 (20.3)	137.5 (15.7)	133.6 (20.6)
Total cholesterol, median(IQR)	4.6 (3.7-5.4)	4.6 (4.0-5.4)	4.3 (3.7-5.0)	4.8 (3.8-5.5)	4.2 (3.6-4.9)	4.2 (3.7-5.0)	4.0 (3.4-4.6)	4.1 (3.5-4.9)
HDL-c, median(IQR)	1.2 (1.1-1.7)	1.3 (1.1-1.5)	1.2 (1.0-1.4)	1.3 (1.0-1.6)	1.1 (1.0-1.4)	1.2 (1.0-1.4)	1.2 (0.9-1.4)	1.2 (0.9-1.5)
LDL-c, median(IQR)	2.3 (1.7-3.1)	2.4 (1.9-3.1)	2.2 (1.6-2.8)	2.6 (1.7-3.0)	2.1 (1.7-2.8)	2.2 (1.7-2.8)	1.9 (1.6-2.5)	2.1 (1.6-2.8)
Triglycerides, median(IQR)	1.9 (1.1-2.4)	1.7 (1.2-2.5)	1.5 (1.0-2.3)	1.5 (1.2-2.1)	1.7 (1.3-2.2)	1.6 (1.2-2.3)	1.6 (1.2-2.1)	1.4 (1.0-1.9)
HbA1c, median(IQR)	39.5 (34.0-48.2)	40.0 (36.0-45.0)	42.0 (36.0-53.0)	39.0 (35.5-44.5)	41.0 (37.0-49.8)	39.0 (36.0-44.0)	43.0 (36.8-58.5)	39.0 (35.0-44.0)
eGFR (CKD-EPI), median(IQR)	85.5 (45.6-99.1)	84.6 (59.8-96.7)	91.6 (73.2-103.1)	84.0 (52.2-98.5)	82.2 (58.5-97.2)	82.7 (51.0-96.2)	77.0 (66.3-95.9)	81.1 (51.4-94.3)
CVD history (yes), n(%)	79 (58.5)	207 (53.2)	83 (52.9)	92 (43.4)	211 (51.1)	233 (48.2)	34 (53.1)	302 (46.3)
Hypertension history (yes), n(%)	2 (1.5)	3 (0.8)	1 (0.6)	3 (1.4)	2 (0.5)	4 (0.8)	1 (1.6)	4 (0.6)
Diabetes (yes), n(%)	13 (9.6)	20 (5.1)	12 (7.6)	6 (2.8)	23 (5.6)	36 (7.5)	9 (14.1)	28 (4.3)

Notes: n = number; % = percentage; IQR = interquartile range; BMI = body mass index; sysBP = systolic blood pressure; HR = heart rate; SD = standard deviation; Hb = haemoglobin; HDL-c = high-density lipoprotein cholesterol; LDL-c = low-density lipoprotein cholesterol; HbA1c = glycated haemoglobin; eGFR CKD-EPI = estimated glomerular filtration rate using the chronic kidney disease epidemiology collaboration equation; CVD = cardiovascular disease.

Table H. Baseline patient characteristics of patients who visited the vascular medicine OPD, by COVID-19 period.

	Reference period, n=263	period before 1st lockdown, n=587	1st lockdown, n=184	Post-1st lockdown, n=187	2nd lockdown, n=356	Post-2nd lockdown, n=390	3rd lockdown, n=67	Post pandemic, n=580
Sex (female), n(%)	147 (55.9)	326 (55.5)	90 (48.9)	117 (62.6)	202 (56.7)	204 (52.3)	35 (52.2)	314 (54.1)
Age, median(IQR)	55.0 (41.5-66.0)	56.0 (44.0-65.0)	52.0 (41.0-62.0)	53.0 (40.0-61.5)	53.5 (45.0-64.0)	53.0 (41.0-63.0)	53.0 (36.5-62.5)	54.0 (40.0-64.0)
Smoker (yes), n(%)	11 (34.4)	12 (13.3)	6 (28.6)	5 (21.7)	13 (24.1)	18 (31.6)	3 (30.0)	25 (32.5)
BMI, median(IQR)	26.3 (23.5-29.6)	26.0 (23.5-29.7)	26.3 (23.7-29.1)	25.8 (23.7-29.3)	26.4 (23.4-29.9)	26.9 (23.8-31.1)	27.2 (24.2-30.5)	26.3 (23.4-29.5)
sysBP, mean(SD)	143.6 (26.6)	144.8 (28.6)	140.6 (24.9)	139.5 (25.9)	148.0 (28.6)	151.1 (29.2)	146.5 (28.4)	145.3 (29.7)
HR, mean(SD)	75.1 (15.3)	73.6 (13.7)	72.6 (12.5)	73.7 (13.3)	74.6 (14.0)	75.0 (15.2)	75.5 (14.2)	73.5 (14.3)
Hb, mean(SD)	142.9 (15.8)	142.0 (15.5)	147.1 (17.8)	143.4 (18.9)	147.1 (16.7)	147.2 (15.2)	141.1 (17.4)	141.0 (17.5)
Total cholesterol, median(IQR)	5.3 (4.5-6.3)	5.2 (4.2-6.2)	4.8 (4.1-6.1)	5.2 (4.3-5.9)	5.1 (4.1-6.2)	5.2 (4.2-6.1)	5.4 (4.5-6.2)	4.9 (4.0-5.7)
HDL-c, median(IQR)	1.4 (1.1-1.6)	1.4 (1.2-1.7)	1.4 (1.1-1.7)	1.4 (1.1-1.7)	1.3 (1.1-1.6)	1.2 (1.0-1.5)	1.3 (1.1-1.5)	1.3 (1.0-1.6)
LDL-c, median(IQR)	3.1 (2.3-4.1)	3.0 (2.2-3.9)	2.8 (2.0-3.7)	2.9 (2.2-3.7)	2.9 (2.2-3.7)	2.9 (2.3-3.8)	3.3 (2.4-4.0)	2.9 (2.1-3.6)
Triglycerides, median(IQR)	1.4 (1.1-2.2)	1.3 (0.9-1.9)	1.4 (1.0-2.1)	1.3 (0.9-2.0)	1.4 (1.0-2.4)	1.5 (1.1-2.6)	1.6 (0.9-2.4)	1.3 (1.0-2.0)
HbA1c, median(IQR)	38.0 (35.0-41.0)	37.0 (34.0-40.0)	37.0 (33.0-40.0)	37.0 (34.0-40.0)	37.0 (35.0-41.0)	37.0 (34.0-40.0)	36.0 (33.5-39.0)	37.0 (34.0-40.0)
eGFR (CKD-EPI), median(IQR)	95.6 (83.6-107.7)	98.0 (87.1-107.9)	99.3 (92.9-111.3)	101.1 (81.8-110.9)	98.9 (85.0-109.4)	100.0 (85.5-111.5)	92.9 (79.1-107.1)	95.5 (82.1-109.0)
CVD history (yes), n(%)	23 (8.7)	49 (8.3)	18 (9.8)	15 (8.0)	40 (11.2)	38 (9.7)	7 (10.4)	89 (15.3)
Hypertension history (yes), n(%)	33 (12.5)	90 (15.3)	24 (13.0)	22 (11.8)	62 (17.4)	68 (17.4)	18 (26.9)	110 (19.0)
Diabetes (yes), n(%)	17 (6.5)	33 (5.6)	8 (4.3)	10 (5.3)	19 (5.3)	16 (4.1)	3 (4.5)	28 (4.8)

Notes: n = number; % = percentage; IQR = interquartile range; BMI = body mass index; sysBP = systolic blood pressure; HR = heart rate; SD = standard deviation; Hb = haemoglobin; HDL-c = high-density lipoprotein cholesterol; LDL-c = low-density lipoprotein cholesterol; HbA1c = glycated haemoglobin; eGFR CKD-EPI = estimated glomerular filtration rate using the chronic kidney disease epidemiology collaboration equation; CVD = cardiovascular disease.
