# Supplementary material for: Comparison of outpatient attendance, cardiovascular risk management and cardiovascular health across preCOVID-19, during and postCOVID-19 periods: a prospective cohort study
Source: BMJ Open. 2025 Jul 16;15(7):e092374. doi: 10.1136/bmjopen-2024-092374 (PMC12273069; doi:10.1136/bmjopen-2024-092374)
Supplement: online supplemental file 4 [file bmjopen-15-7-s004.pdf]

Supplement 4

Table I. Quantification of the median number of first appointments per week per COVID period, in total and per OPD.

	**Reference period**	**Pre-1st lockdown**	**1st lockdown**	**Post-1st lockdown**	**2nd lockdown**	**Post-2nd lockdown**	**3rd lockdown**	**Post-pandemic**
	Median(IQR)	Median (IQR)	Median(IQR)	Median(IQR)	Median(IQR)	Median(IQR)	Median(IQR)	Median(IQR)
Total	99 (85-111)	107 (95-115)	63 (55-72)	70 (63-82)	68 (59-75)	71 (61-78)	71 (59-76)	69 (59-75)
Clinical OPD								
- Cardiology	57 (48-63)	56 (46-60)	30 (28-33)	21.5 (18-28)	20 (17-26)	25 (22-29)	26 (24-28)	27 (23-31)
- Geriatrics	3 (2-4)	9 (6-10)	7.5 (5-9)	8 (5-11)	6 (4-7)	6 (4-7)	5 (5-6)	4 (3.5-5)
- Diabetology	7 (5-9)	8 (5-9)	5 (4-6)	6.5 (4-8)	6 (4-7)	7 (5-8)	7 (5-8)	5 (3.7-7)
- Nephrology	7 (6-10)	7 (5-8)	3 (2-5)	5 (3-6)	5 (3-7)	5 (4-8)	5 (5-6)	4 (3-6)
- Vascular medicine	15 (12-18)	17 (16-19)	10 (7-13)	13.5 (11-14)	13 (11-16	11 (9-14)	11.5 (10-12)	11 (10-14)
- Multidisciplinary vascular surgery	7 (5-10)	11 (6-14)	11 (6-14)	9 (8-11)	13.5 (12-17)	15 (13-17)	12 (6-14)	13 (11-17)

Notes: IQR = interquartile range; OPD = outpatient department.
