# Supplementary figures and images for: Comparison of outpatient attendance, cardiovascular risk management and cardiovascular health across preCOVID-19, during and postCOVID-19 periods: a prospective cohort study
Source: BMJ Open. 2025 Jul 16;15(7):e092374. doi: 10.1136/bmjopen-2024-092374 (PMC12273069; doi:10.1136/bmjopen-2024-092374)

## Supplement 5

Figure A. Follow-up appointments by type of appointment, by OPD.

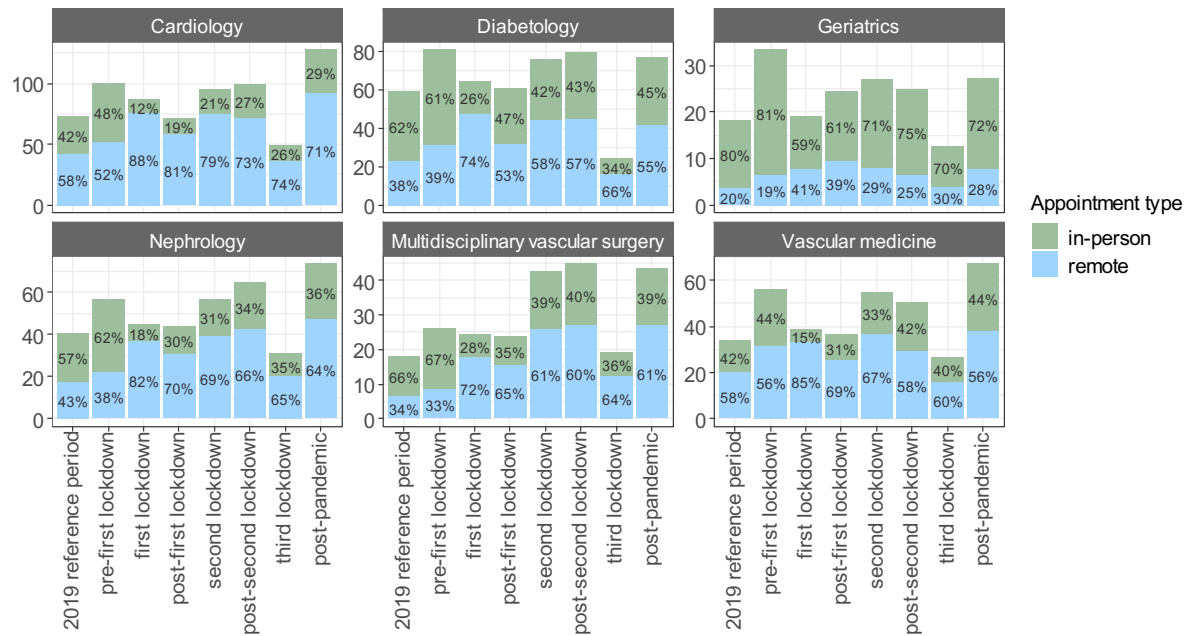

Supplement: online supplemental file 5 [file bmjopen-15-7-s005.pdf]
