# Supplementary material for: Comparison of outpatient attendance, cardiovascular risk management and cardiovascular health across preCOVID-19, during and postCOVID-19 periods: a prospective cohort study
Source: BMJ Open. 2025 Jul 16;15(7):e092374. doi: 10.1136/bmjopen-2024-092374 (PMC12273069; doi:10.1136/bmjopen-2024-092374)
Supplement: online supplemental file 6 [file bmjopen-15-7-s006.pdf]

Supplement 6

Table J. Comparison of the CVRM indicator extractability at baseline across COVID-19 periods.

	**Ref. period, %**	**Pre-1st lockdown, %**	**1st lockdown, %**	**Post-1st lockdown, %**	**2nd lockdown, %**	**Post-2nd lockdown, %**	**3rd lockdown, %**	**Post-pandemic, %**	***P***	***P****
BMI	50	50	43	52	57	50	48	47	.07	<.01
Smoking	11	12	10	11	12	11	13	9	.44	<.01
HR	45	49	36	44	50	41	39	38	.84	<.01
sysBP	54	54	43	54	59	51	50	47	.75	<.01
Hb	59	63	51	60	63	59	61	57	.68	<.01
Tot-c	39	43	30	39	41	35	34	36	.07	<.01
HDL-c	39	42	29	38	41	34	34	35	.045	<.01
LDL-c	39	42	29	38	40	34	34	35	.05	<.01
Trig	39	43	30	39	41	34	35	36	.08	<.01
HbA1c	33	38	30	38	38	36	36	36	.06	<.01
eGFR	63	65	54	66	66	63	64	60	.48	<.01

Notes: % = percentage; *P* = probability value; ref = reference; BMI = body mass index; HR = heart rate; sysBP = systolic blood pressure; Hb = haemoglobin; Tot-c = total cholesterol; HDL-c = high-density lipoprotein cholesterol; LDL-c = low-density lipoprotein cholesterol; Trig = triglycerides; HbA1c = glycated haemoglobin; eGFR = estimated glomerular filtration rate using the chronic kidney disease epidemiology collaboration equation. * = p-value of the interaction between sex and the COVID-19 period, estimated using multivariable logistic regression. ** = p-value of the interaction between OPD and COVID-19 period, estimated using multivariable logistic regression.

Figure B. Extractability of CVRM indicators across COVID-19 periods, by OPD.

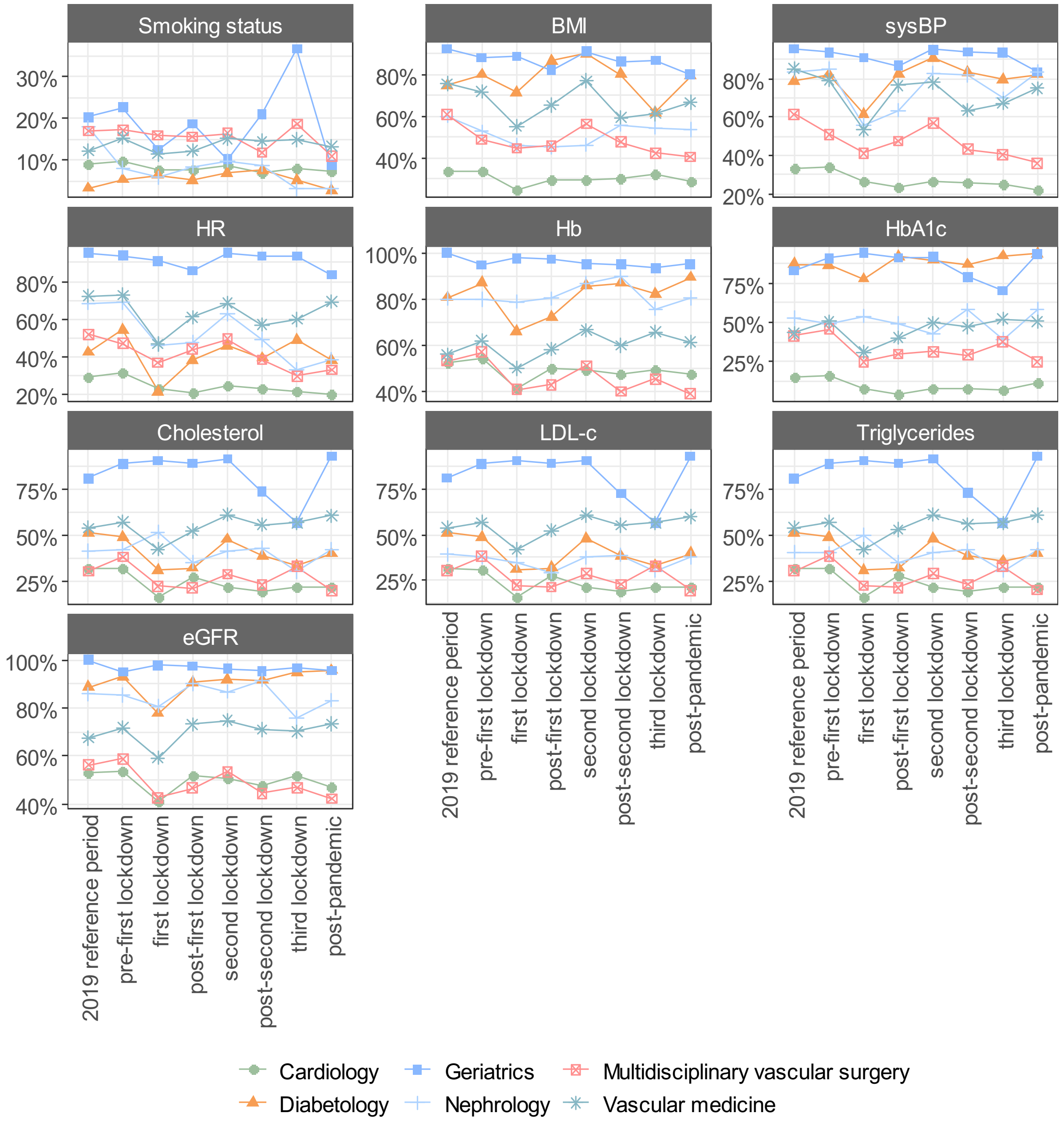

Notes: BMI = body mass index; sysBP = systolic blood pressure; HR = heart rate; Hb = haemoglobin; HbA1c = glycated haemoglobin; LDL-c = low-density lipoprotein cholesterol; eGFR = estimated glomerular filtration rate using the chronic kidney disease epidemiology collaboration equation.
