# Supplementary material for: Comparison of outpatient attendance, cardiovascular risk management and cardiovascular health across preCOVID-19, during and postCOVID-19 periods: a prospective cohort study
Source: BMJ Open. 2025 Jul 16;15(7):e092374. doi: 10.1136/bmjopen-2024-092374 (PMC12273069; doi:10.1136/bmjopen-2024-092374)
Supplement: online supplemental file 7 [file bmjopen-15-7-s007.pdf]

Supplement 7

Table K. Sensitivity analysis into the association between period of the first appointment and CVH, adjusted for covariates*, stratified by OPD (after multiple imputation).

	**Cardiology**	**Vascular medicine**	**Nephrology**	**Geriatrics**	**Diabetology**	**Multidisciplinary vascular surgery**
	Beta (95% CI)	Beta (95% CI)	Beta (95% CI)	Beta (95% CI)	Beta (95% CI)	Beta (95% CI)
Intercept	69.70 (63.10 - 76.30)	74.03 (69.62 - 78.43)	73.29 (67.12 - 79.46)	60.22 (50.90 - 69.53)	86.34 (81.36 - 91.33)	63.75 (57.09 - 70.40)
COVID-19 period						
- reference period	Ref.	Ref.	Ref.	Ref.	Ref.	Ref.
- pre-1st lockdown	2.12 (-0.05 - 4.29)	4.57 (1.24 - 7.89)	-2.38 (-7.48 - 2.72)	0.36 (-4.38 - 5.10)	3.46 (-1.04 - 7.96)	4.08 (-1.19 - 9.35)
- 1st lockdown	-0.67 (-4.60 - 3.27)	5.08 (0.71 - 9.45)	9.85 (2.25 - 17.46)	6.09 (0.47 - 11.72)	4.32 (-1.36 - 9.99)	1.33 (-5.56 - 8.23)
- post-1st lockdown	3.78 (-3.24 - 10.81)	3.41 (-1.07 - 7.88)	5.48 (-1.76 - 12.73)	3.16 (-2.25 - 8.56)	4.44 (-1.09 - 9.98)	1.55 (-4.10 - 7.21)
- 2nd lockdown	0.87 (-4.24 - 5.97)	1.39 (-2.18 - 4.96)	-1.93 (-7.56 - 3.71)	3.67 (-1.39 - 8.74)	-0.57 (-5.46 - 4.32)	-0.79 (-5.53 - 3.95)
- post-2nd lockdown	3.41 (1.12 - 5.70)	-0.08 (-3.61 - 3.45)	1.65 (-3.58 - 6.87)	3.12 (-1.82 - 8.05)	3.25 (-1.39 - 7.89)	4.57 (-0.66 - 9.80)
- 3rd lockdown	4.32 (-8.27 - 16.91)	1.62 (-4.49 - 7.73)	1.04 (-8.55 - 10.63)	0.63 (-6.93 - 8.19)	-4.83 (-12.46 - 2.80)	-2.98 (-10.24 - 4.28)
- back to normal	2.11 (-1.56 - 5.77)	3.70 (0.32 - 7.08)	4.82 (-0.46 - 10.10)	6.91 (1.99 - 11.82)	5.49 (0.99 - 9.99)	4.18 (-0.64 - 9.01)

Notes: beta = beta coefficient indicating the difference in CVH score between the periods during the COVID-19 pandemic and the reference period; the green cells indicate a significant increase in CVH score (i.e., better CVH); CI = confidence interval. *Adjusted for age, biological sex, cardiovascular disease history, and whether the patient had an appointment before the study period.
