# Supplementary material for: Comparison of outpatient attendance, cardiovascular risk management and cardiovascular health across preCOVID-19, during and postCOVID-19 periods: a prospective cohort study
Source: BMJ Open. 2025 Jul 16;15(7):e092374. doi: 10.1136/bmjopen-2024-092374 (PMC12273069; doi:10.1136/bmjopen-2024-092374)
Supplement: Supplementary file 8 [file bmjopen-2024-092374.reviewer_comments.pdf]

PEER REVIEW HISTORY

BMJ Open publishes all reviews undertaken for accepted manuscripts. Reviewers are asked to complete a checklist review form and are provided with free text boxes to elaborate on their assessment. These free text comments are reproduced below.

ARTICLE DETAILS

Title (Provisional)

Comparison of outpatient attendance, cardiovascular risk management, and cardiovascular health across pre-, during, and post-COVID-19 periods: a prospective cohort study

Authors

Zondag, Anna G.M.; Haitjema, Saskia; de Groot, Mark C.H.; de Boer, Annemarijn R; van Solinge, Wouter W; Bots, Michiel L; Vernooij, Robin W.M.

VERSION 1 - REVIEW

**Reviewer**	**1**
**Name**	**Choudhary, Rahul**
**Affiliation**	**All India Institute of Medical Sciences, Department of Cardiology**
**Date**	**05-Dec-2024**
**COI**	**None**

I would like to commend the authors for addressing this issue, as it paves the way for the development of appropriate policies in the future.

Including precise data on emergency admissions and MACE among patients would have further strengthened the study, in addition to the decrease in OPD numbers.

**Reviewer**	**2**
**Name**	**Amin, Mohammad Ashraful**
**Affiliation**	**North South University, Public health**
**Date**	**04-Apr-2025**
**COI**	**None**

---

1. Title

The title is informative but could be more concise. Consider trimming excess words or restructuring for improved clarity.

2. Abstract: The abstract presents objectives, methods, and key findings well, but the conclusion could be more impactful by specifying key implications for policy or practice.

3. Introduction

Comment: Consider streamlining the introduction slightly. A few sections are repetitive, especially around the description of the GTFCC strategy.

4. Methods

Comment: The methodology is robust and clearly described, but more detail could be given on:

The rationale for choosing countries.

Any limitations of qualitative data analysis techniques used.

Whether data saturation was achieved in qualitative interviews.

5. Results

Consider: Adding clearer sub-headings within large sections for easier navigation.

Highlighting any significant themes from qualitative interviews using short quotations or summaries in a table.

6. Discussion: The discussion is detailed but could benefit from:

Tighter linkage between findings and GTFCC strategic goals.

A more critical reflection on implementation gaps and potential contextual barriers.

Reference to global health literature beyond the GTFCC to broaden the context.

7. Strengths and Limitations

This section is honest and informative. However, you may expand briefly on how findings might be applicable to other LMICs or global health programmes.

8. Policy Implications

Well-structured, but it may help to present this as bullet points or in a table to increase clarity for decision-makers.

9. Formatting and Language

Minor grammatical inconsistencies and overly long sentences are present. A final language edit for conciseness and clarity is recommended.

10. References: can be take some information of the Asia based from this paper :PMCID: PMC9302201 PMID: 35873273 - for comparison during COVID-19 times.

**Reviewer**	**3**
**Name**	**Tran, Luong**
**Affiliation**	**Woolcock Institute of Medical Research, Faculty of Medicine and Health**
**Date**	**17-Apr-2025**
**COI**	**None**

The abstract is well-written, with clear objectives, outcomes and conclusions. The last sentence (lines 51-52) is about an implication, but it may not be 'genuinely correct' in the context of COVID-19, especially during the early stage of the pandemic. People with underlying health conditions, like CVDS, were reported associated with a higher risk of COVID-19 and have higher mortality risk. So, this implication should be removed or revised.

The introduction is clear and well-written.

Methods: I have no concerns about statistical analysis and methods used. Below is the only concern.

Although authors claim that the timelines for determinants are based on 'the Dutch National Institute of Public Health throughout the COVID-19', which is still unclear for international readers. The COVID-19 pandemic began around December 2019, this time point should be used as a reference, and then the before first lockdown, continues for other periods. Post-COVID-19 pandemic may vary among countries. However, before the pandemic period should be clearly defined. For this reason, I am concerned about the dates of periods being divided to have better results (which do not need to be correct), which is what readers might interpret. I would recommend authors reconsider this point.

Findings: Findings were reported clearly and understandable. As I mentioned in the method section about the division of comparable periods, results might be different.

VERSION 1 - AUTHOR RESPONSE

Reviewer: 1
Dr. Rahul Choudhary, All India Institute of Medical Sciences

Comments to the Author:

I would like to commend the authors for addressing this issue, as it paves the way for the development of appropriate policies in the future.
Including precise data on emergency admissions and MACE among patients would have further strengthened the study, in addition to the decrease in OPD numbers.

RESPONSE: We thank the reviewer for the kind words, for taking the time to review our manuscript and for the valuable feedback.

We acknowledge that including information on emergency admission and the occurrence of MACE among patients will be interesting to further investigate the impact of the COVID-19 pandemic. Yet this was beyond the scope of our current study. Furthermore, to date, more scientific research has already been conducted in the Netherlands and in other countries on the impact of the COVID-19 pandemic on hospital admissions for acute CVD (1-5) and emergency department utilisation (6,7), while less is known on its impact on patients with or at risk of CVD attending OPDs for prevention purposes. We do, however, emphasise in the Discussion of our manuscript that, among other things, further study is needed to study whether the patients that were not able or avoided the OPD more often suffered from events. It reads:

- *"Furthermore, further study is warranted to evaluate how remote appointments may have influenced the patient's prognosis. As important would be to study whether the patients with worse CVH, who avoided or were unable to attend OPDs, indeed more often suffered from events that could have been prevented."*

References:

1. Kiss P, Carcel C, Hockham C, Peters SAE. The impact of the COVID-19 pandemic on the care and management of patients with acute cardiovascular disease: A systematic review. Eur Heart J Qual Care Clin Outcomes. 2021;7(1):18–27.
2. Rinkel LA, Prick JCM, Slot RER, Sombroek NMA, Burggraaff J, Groot AE, et al. Impact of the COVID-19 outbreak on acute stroke care. J Neurol. 2021;268(2):403-408.
3. Mafham MM, Spata E, Goldacre R, Gair D, Curnow P, Bray M, et al. COVID-19 pandemic and admission rates for and management of acute coronary syndromes in England. Lancet. 2020;396(10248):381–9.
4. Kempers EK, Chen Q, Visser C, van Gorp ECM, Klok FA, Cannegieter SC, et al. Changes in incidence of hospitalization for cardiovascular diseases during the COVID-19 pandemic in The Netherlands in 2020. Sci Rep. 2023;13(1):12832.
5. Khan Y, Verhaeghe N, Devleesschauwer B, et al. Impact of the COVID-19 pandemic on delayed care of cardiovascular diseases in Europe: a systematic review. Lancet. 2023;402(1):S61.
6. Barten DG, Latten GHP, van Osch FHM. Reduced Emergency Department Utilization During the Early Phase of the COVID-19 Pandemic: Viral Fear or Lockdown Effect? Disaster Medicine and Public Health Preparedness. 2022;16(1):36–9. doi:10.1017/dmp.2020.303
7. Gutovitz S, Pangia J, Finer A, Rymer K, Johnson D. Emergency Department Utilization and Patient Outcomes During the COVID-19 Pandemic in America. J Emerg Med. 2021;60(6):798-806.

Reviewer: 2

Dr. Mohammad Ashraful Amin, North South University

We thank the reviewer for taking the time to review our manuscript. We have used the comments to further improve our manuscript accordingly. Please see our point-by-point responses to the comments below. However, for some of the comments we would like to ask for clarification. Some comments refer to the "GTFCC strategy", which we assumed to be an abbreviation for the "Global Task Force on Cholera Control". It is not entirely clear to us, however, how the GTFCC relates specifically to our study, which compared outpatient care utilisation, cardiovascular risk management and

cardiovascular health before, during and after the COVID-19 pandemic in the Netherlands. Nevertheless, we hope that the revisions we made sufficiently improved our manuscript.

Comments to the Author:

1. Title

The title is informative but could be more concise. Consider trimming excess words or restructuring for improved clarity.

RESPONSE: We thank the reviewer for the comment. We have adjusted the title. It now reads:

- *"Comparison of outpatient attendance, cardiovascular risk management, and cardiovascular health across pre-, during, and post-COVID-19 periods: a prospective cohort study"*

2. Abstract: The abstract presents objectives, methods, and key findings well, but the conclusion could be more impactful by specifying key implications for policy or practice.

RESPONSE: We have changed the conclusion in the abstract substantially and it now reads:

- *"During the COVID-19 pandemic, weekly numbers of first appointments to OPDs decreased and a population with a higher CVH score (i.e., better CVH) visited certain OPDs, especially during the first lockdown period. This suggests that patients with poorer CVH more often avoided or were unable to visit OPDs, which might have resulted in missed opportunities to control cardiovascular risk factors and potentially may have led to preventable disease outcomes. For future epidemics and pandemics, it seems vital to develop a strategy that includes an emphasis on seeking healthcare when needed, with specific attention to patients at higher CVD risk."*

3. Introduction

Comment: Consider streamlining the introduction slightly. A few sections are repetitive, especially around the description of the GTFCC strategy.

RESPONSE: We have revised the introduction accordingly.

4. Methods

Comment: The methodology is robust and clearly described, but more detail could be given on:

The rationale for choosing countries.

Any limitations of qualitative data analysis techniques used.

Whether data saturation was achieved in qualitative interviews.

RESPONSE: We thank the reviewer for the kind words. While we completely agree with the reviewer that data saturation is of major importance to mention and discuss in qualitative research studies, we would like to note that our study contained quantitative data collected in the Netherlands only. Therefore, neither data saturation nor the limitations of the qualitative data analysis techniques were discussed in the methods section. The limitations of our study, including the limitations related to the (re)use of routine care data for research purposes, are discussed in the 'Limitations' section of the discussion (page 15 of the revised manuscript). We hope to have provided the reviewer with sufficiently detailed information about our study's limitations.

5. Results
5.1 Consider: Adding clearer sub-headings within large sections for easier navigation.

<u>RESPONSE</u>: We have now added additional sub-headings to the Results, in addition to the ones already present. The newly added sub-headings are "First appointments over time, overall and by OPD" and "Follow-up appointments over time, overall and by OPD".

5.2 Highlighting any significant themes from qualitative interviews using short quotations or summaries in a table.

<u>RESPONSE:</u> We would like to kindly refer the reviewer to our response to comment #4.

6. Discussion: The discussion is detailed but could benefit from:
Tighter linkage between findings and GTFCC strategic goals.
A more critical reflection on implementation gaps and potential contextual barriers.
Reference to global health literature beyond the GTFCC to broaden the context.

<u>RESPONSE</u>: We have revised the discussion and would like to invite the reviewer to elaborate on how GTFCC relates to our article.

7. Strengths and Limitations
This section is honest and informative. However, you may expand briefly on how findings might be applicable to other LMICs or global health programmes.

<u>RESPONSE</u>: We thank the reviewer for this suggestion. We have added the following sentences to the limitations section of our study:

- *"…, although COVID-19 was a global pandemic, our study focused exclusively on its impact within a tertiary care centre in the Netherlands. The extrapolation of our results to other countries may be challenging, given the variability of the COVID-19 pandemic situation and the restrictive measures imposed across countries and between healthcare settings."*

8. Policy Implications
Well-structured, but it may help to present this as bullet points or in a table to increase clarity for decision-makers.

<u>RESPONSE</u>: We have provided this according to the format of BMJ Open. Hence, we respectfully would like to keep it as such.

9. Formatting and Language
Minor grammatical inconsistencies and overly long sentences are present. A final language edit for conciseness and clarity is recommended.

<u>RESPONSE</u>: We thank the reviewer for the feedback. We have carefully revised the manuscript to address the grammatical inconsistencies and have edited overly long sentences throughout the text.

10. References: can be take some information of the Asia based from this paper :PMCID: PMC9302201 PMID: 35873273 - for comparison during COVID-19 times.

RESPONSE: We thank the reviewer for providing this reference, we have read it with great interest. Although the article provides valuable insights into the prevalence of anxiety and depressing among hospitalised cardiovascular patients between March to June 2021 (i.e., during the COVID-19 pandemic), it does not describe whether there was a difference in the prevalence of anxiety and depression during the COVID-19 pandemic compared to a reference period before the COVID-19 pandemic. Therefore, we feel unable to relate the findings of this article to our results.

Reviewer: 3
Dr. Luong Tran, Woolcock Institute of Medical Research

We would like to thank the reviewer for their valuable time to thoroughly review our manuscript. We have addressed the comments point-by-point below.

Comments to the Author:
1. The abstract is well-written, with clear objectives, outcomes and conclusions. The last sentence (lines 51-52) is about an implication, but it may not be 'genuinely correct' in the context of COVID-19, especially during the early stage of the pandemic. People with underlying health conditions, like CVDS, were reported associated with a higher risk of COVID-19 and have higher mortality risk. So, this implication should be removed or revised.

RESPONSE: We agree that, based on our study, we cannot be certain whether it led to preventable disease outcomes, which is why we used modal auxiliary verbs such as 'may' and 'might' to express our uncertainty. Additionally, in the Discussion section of our manuscript, we recommend future studies to investigate this. It reads:

- *"As important would be to study whether the patients with worse CVH, who avoided or were unable to attend OPDs, indeed more often suffered from events that could have been prevented."*

2. The introduction is clear and well-written.

RESPONSE: We thank the reviewer for the kind words.

3. Methods: I have no concerns about statistical analysis and methods used. Below is the only concern. Although authors claim that the timelines for determinants are based on 'the Dutch National Institute of Public Health throughout the COVID-19', which is still unclear for international readers. The COVID-19 pandemic began around December 2019, this time point should be used as a reference, and then the before first lockdown, continues for other periods. Post-COVID-19 pandemic may vary among countries. However, before the pandemic period should be clearly defined. For this reason, I am concerned about the dates of periods being divided to have better results (which do not need to be correct), which is what readers might interpret. I would recommend authors reconsider this point.

RESPONSE: We thank the reviewer for voicing this concern and for giving us the opportunity to elaborate on this matter.

During the COVID-19 pandemic, the role of the Dutch National Institute for Public Health and the Environment was, among others, to monitor and report on the spread of the disease outbreak, to

provide scientific advice to the government on the measures to impose, to coordinate the national vaccination program and to issue public health guidelines.(1) Thus, the National Institute for Public Health and the Environment had a significant role. They also initiated and maintained a timeline that kept track of the developments, guidelines and measures implemented during the pandemic in the Netherlands, which is the timeline we used to define the different periods in our study. We agree with the reviewer that background information on the role of the Dutch National Institute for Public Health and the Environment was lacking from our manuscript. Therefore, for clarification purposes, we have now added information on the role of the national institute to the Methods section:

- *"During the COVID-19 pandemic, the role of the Dutch National Institute for Public Health and the Environment was, among others, to monitor and report on the spread of the disease, to provide scientific advice to the government on possible measure to reduce transmission, and to issue public health guidelines.(16)"*

We agree with the reviewer that the timeline of the COVID-19 pandemic likely differed between countries. As such, the dates we used to define each period in our study may not correspond to the exact same phase of the COVID-19 pandemic in other countries (e.g. the lockdowns may have been imposed earlier or later, or lasted for different durations, depending on the national situation). Additionally, the restrictive measures implemented in the Netherlands throughout the COVID-19 pandemic probably differed from those in other countries. Therefore, to provide the readers with more contextual information, we summarised the main restrictions in a timeline (Figure 1). Furthermore, we have now also added the following sentences to the limitations section of our discussion, to inform readers of the potential difficulty of extrapolating our findings to other countries and settings:

- *"…, although COVID-19 was a global pandemic, our study focused exclusively on its impact within a tertiary care centre in the Netherlands. The extrapolation of our results to other countries may be challenging, given the variability of the COVID-19 pandemic situation and the restrictive measures imposed across countries and between healthcare settings."*

However, we respectfully disagree with the reviewer's suggestion that December 2019 should be used as reference for our study. In the Netherlands, the first COVID-19 infection was confirmed in February 2020 (2), and the WHO declared COVID-19 as a pandemic in March 2020 (3), which was also the month in which the first lockdown was imposed in the Netherlands. As we acknowledged in the Discussion (under limitations) of our manuscript, it is indeed debatable whether the periods defined are comparable as seasonality can play a role in, for example, the number of appointments scheduled (e.g., much less during Christmas holidays). To mitigate this and to ensure that we could make a valid comparison between the reference period and one of the lockdown periods, we defined a reference period in 2019 that was truly comparable to the period of the first lockdown.

References:

1. National Institute for Public Health and the Environment [Internet]. [cited 2025 May 21]. Infectious Disease Control. Available from: https://www.rivm.nl/en/infectious-disease-control

2. Visscher K, Kouwenberg LHJA, Oosterhoff M, Rotteveel AH, de Wit GA. Postponed healthcare in The Netherlands during the COVID-19 pandemic and its impact on self-reported health. Front Health Serv. 2023 Jun 23;3:1181532.

3. WHO. WHO Director-General's opening remarks at the media briefing on COVID-19 - 11 March 2020. 2020. Available from: https://www.who.int/director-general/speeches/detail/who-director-general-s-opening-remarks-at-the-media-briefing-on-covid-19---11-march-2020

4. Findings: Findings were reported clearly and understandable. As I mentioned in the method section about the division of comparable periods, results might be different.

RESPONSE: We hope that our response to comment #3 and the revisions made sufficiently addressed the reviewer's concern.

VERSION 2 - REVIEW

Reviewer **2**

Name **Amin, Mohammad Ashraful**

Affiliation **North South University, Public health**

Date **07-Jun-2025**

COI

---

thanks for address all comments.

Reviewer **3**

Name **Tran, Luong**

Affiliation **Woolcock Institute of Medical Research, Faculty of Medicine and Health**

Date **15-Jun-2025**

COI

I'm satisfied that the authors have appropriately addressed all comments. I recommend this work for publication.
